# eNOS S-nitrosylates β-actin on Cys374 and regulates PKC-θ at the immune synapse by impairing actin binding to profilin-1

**DOI:** 10.1371/journal.pbio.2000653

**Published:** 2017-04-10

**Authors:** Almudena García-Ortiz, Noa B. Martín-Cofreces, Sales Ibiza, Ángel Ortega, Alicia Izquierdo-Álvarez, Antonio Trullo, Víctor M. Victor, Enrique Calvo, Begoña Sot, Antonio Martínez-Ruiz, Jesús Vázquez, Francisco Sánchez-Madrid, Juan M. Serrador

**Affiliations:** 1 Dpt. Biología Celular e Inmunología, Centro de Biología Molecular “Severo Ochoa” (CBMSO), CSIC-UAM, Madrid, Spain; 2 Servicio de Inmunología. Hospital Universitario de la Princesa, Universidad Autónoma de Madrid, Instituto de Investigación Sanitaria Princesa (IP), Madrid, Spain; 3 Vascular Pathophysiology Area, Centro Nacional de Investigaciones Cardiovasculares (CNIC), Madrid, Spain; 4 Centro de Investigación Biomédica en Red de Enfermedades Cardiovasculares (CIBERCV), Spain; 5 Immunobiology Unit, Instituto de Medicina Molecular, Faculdade de Medicina de Lisboa, Lisbon, Portugal; 6 Dpt. de Fisiología, Universitat de Valencia, Burjassot, Spain; 7 Unidad de Microscopía, CNIC, Madrid, Spain; 8 Center of Experimental Imaging, Ospedale San Raffaele, Milan, Italy; 9 Servicio de Endocrinología y Nutrición, Hospital Universitario Dr. Peset Aleixandre, Fundación para la Promoción de la Investigación Sanitaria y Biomédica de la Comunidad Valenciana (FISABIO), Valencia, Spain; 10 Laboratorio de Proteómica Cardiovascular, CNIC, Madrid, Spain; 11 Fundación IMDEA-Nanociencia, Madrid, Spain; 12 Centro Nacional de Biotecnología (CNB-CSIC)-IMDEA Nanociencia Associated Unit, Madrid, Spain; Beatson Institute for Cancer Research, United Kingdom of Great Britain and Northern Ireland

## Abstract

The actin cytoskeleton coordinates the organization of signaling microclusters at the immune synapse (IS); however, the mechanisms involved remain poorly understood. We show here that nitric oxide (NO) generated by endothelial nitric oxide synthase (eNOS) controls the coalescence of protein kinase C-θ (PKC-θ) at the central supramolecular activation cluster (c-SMAC) of the IS. eNOS translocated with the Golgi to the IS and partially colocalized with F-actin around the c-SMAC. This resulted in reduced actin polymerization and centripetal retrograde flow of β-actin and PKC-θ from the lamellipodium-like distal (d)-SMAC, promoting PKC-θ activation. Furthermore, eNOS-derived NO S-nitrosylated β-actin on Cys374 and impaired actin binding to profilin-1 (PFN1), as confirmed with the transnitrosylating agent S-nitroso-L-cysteine (Cys-NO). The importance of NO and the formation of PFN1-actin complexes on the regulation of PKC-θ was corroborated by overexpression of PFN1- and actin-binding defective mutants of β-actin (C374S) and PFN1 (H119E), respectively, which reduced the coalescence of PKC-θ at the c-SMAC. These findings unveil a novel NO-dependent mechanism by which the actin cytoskeleton controls the organization and activation of signaling microclusters at the IS.

## Author summary

T cells are an essential arm of the immunity against the invasion of pathogenic agents in organisms. These specialized cells recognize foreign antigens displayed on the surface of antigen-presenting cells (APC) by means of the T cell receptor (TCR). Early signaling takes place in these cells through the specific clustering of TCRs, which trigger the recruitment of signaling molecules to the immune synapse (IS), a plasma membrane–associated intercellular domain important for T cell activation. In this location, several signaling molecules that include the protein kinase C-θ (PKC-θ) form microclusters that are translocated centripetally towards the center of the IS, following the retrograde movement of actin. In this study, we show that nitric oxide (NO) formed by endothelial nitric oxide synthase (eNOS) regulates the translocation of PKC-θ to the IS, increasing its activation. eNOS can effectively modify β-actin by S-nitrosylation on Cys374, reducing its ability to bind profilin-1 (PFN1)—a protein required for actin polymerization—polymerize and flow from the periphery to the central region of the IS. We propose that eNOS-derived NO controls actin polymerization via S-nitrosylation of actin as one of the major driving forces for the transport of PKC-θ towards the central area of the IS, which is essential for T cell activation.

## Introduction

Nitric oxide (NO) is a free radical that is highly reactive against transition metals in prosthetic groups, Cys, and Tyr of proteins whose activity can be regulated by nitrosylation and nitration [1,2]. As a signaling molecule, NO exerts important regulatory functions in T cells [3]. In mice, low levels of NO favor T helper 1 (Th1) differentiation through a cyclic guanosine monophosphate (cGMP)-dependent mechanism, whereas high levels of NO from inducible nitric oxide synthase (iNOS) prevents Th1 and Th17 responses [4,5]. Conversely, in humans, the induction and stability of Th17 responses have been associated with iNOS-produced NO [6], making evident the functional differences described for NO between the murine and human systems [7].

In steady-state conditions, human T cell lines and primary T lymphocytes express endothelial (e)NOS, which is chiefly localized on the microtubule-organizing center (MTOC)-associated Golgi, producing NO upon T cell receptor (TCR)-triggering and providing pro-activatory signals at the immune synapse (IS) [8,9], a plasma membrane–associated intercellular compartment of adhesion and signaling found in T cells during their antigen-specific interactions with antigen-presenting cells (APCs) [10,11]. The IS is organized in a central area or supramolecular activation cluster (c-SMAC) in which the signaling molecule CD3 concentrates, a ring-shaped peripheral (p)-SMAC where the β2 integrin LFA-1 clusters with talin and actin, and a lamellipodium-like distal (d)-SMAC characterized by a ring of F-actin [12]. Among its functions, the IS modulates T cell activation, acting as a rheostat to attenuate or sustain signaling by degradation of TCR–ligand complexes at the c-SMAC or positioning signaling microclusters at the p-SMAC, respectively. [13–15]

Protein kinase C-θ (PKC-θ), a novel protein kinase C preferentially expressed in T lymphocytes, activates nuclear factor kappa B (NF-κB) through phosphorylation of CARD-containing MAGUK protein 1 (CARMA1) and assembly of the CARMA1–BCL10–MALT1 complex, participates in the differentiation of Th2 and Th17 cells [16], localizes at the c-SMAC, and is involved in the translocation of the MTOC towards the IS where PKC-θ forms a ring-shaped area of signaling near the p-SMAC [17,18]. The localization and activation of PKC-θ at the IS depends on several factors, including its association with diacylglycerol (DAG) and CD28, phosphorylation on Thr538 by the MAP4K germinal center kinase-like kinase (GLK), sumoylation, and the reorganization of actin [19–23].

In T cells, actin allows TCR triggering, supports receptor-mediated intercellular adhesion, maintains the association of signaling microclusters, and organizes the spatial distribution of signaling receptors, adaptors, and kinases at the IS by centripetal retrograde flow-mediated transport of microclusters towards the c-SMAC, a process achieved by actin treadmilling at the d-SMAC [13,24,25]. Although a large body of evidence points to the actin cytoskeleton as the main organizer of signaling molecules at the IS, besides findings based on either pharmacological agents disturbing actin dynamics or the interference on the function of the actin nucleator Arp2/3 and its regulation by nucleation-promoting elements such as WAVE2 and Wiskott-Aldrich syndrome protein (WASP) [26–29], little is known about how β-actin itself takes part in the mechanisms that orchestrate the flow of signaling microclusters towards the c-SMAC.

We have previously shown that eNOS impairs the clustering of CD3 at the c-SMAC [8], suggesting that NO may regulate the organization and activation of signaling molecules at the IS. In this work, we have investigated the mechanism by which NO may exert this regulation. Focusing on PKC-θ as a c-SMAC hallmark, we show that eNOS-derived NO controls the organization and activation of PKC-θ at the IS, a result confirmed in studies using the NO donor, S-nitroso-L-cysteine (Cys-NO). In antigen-specific T cell-APC conjugates, eNOS translocated to the IS, colocalizing with F-actin, fostering actin depolymerization, and reducing actin retrograde and PKC-θ inward flow from the lamellipodium-like d-SMAC. Mechanistic insights are provided by demonstrating that NO regulates the organization of PKC-θ by S-nitrosylation of β-actin on Cys374, a posttranslational modification that, disturbing the binding of actin to PFN1, controls the organization of the actin cytoskeleton from the lamellipodium-like d-SMAC.

## Results

### eNOS regulates the recruitment and localization of PKC-θ at the IS

**Fig 1:**
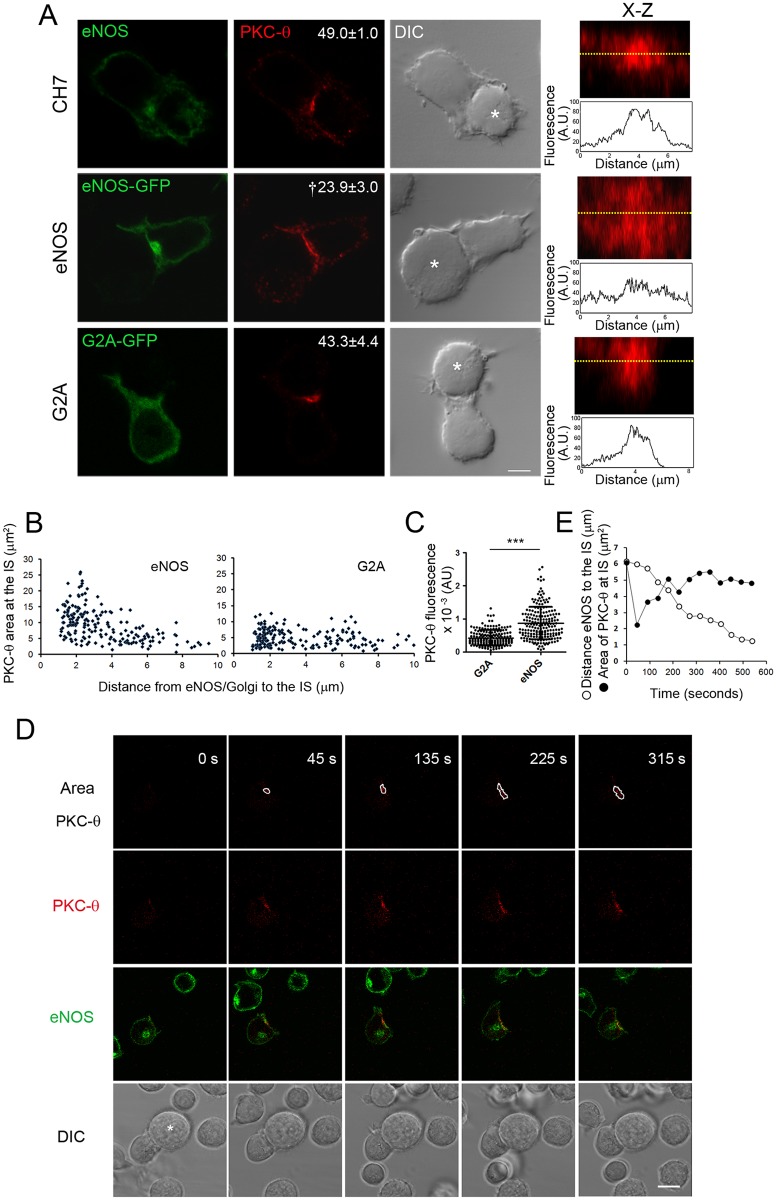
Endothelial Nitric Oxide Synthase (eNOS) overexpression disturbs the coalescence of Protein Kinase C-θ (PKC-θ) at the central Supramolecular Activation Cluster (c-SMAC). (A) Localization of PKC-θ (red) and eNOS, eNOS-green fluorescent protein (GFP), or G2A-GFP (green) in the immune synapse (IS) of CH7C17, eNOS, and G2A T cells conjugated for 20 min with staphylococcal enterotoxin B (SEB)-pulsed Raji antigen-presenting cells (APCs) (asterisks). Bar = 4 μm. Percentages of cells with PKC-θ concentrated at the c-SMAC are indicated as mean ± standard error of the mean (SEM). *n* = 4. † *p* ≤ 0.01. On the right, X–Z plane projections of PKC-θ at the IS and fluorescence profiles along the yellow dotted lines are shown. (B) Area occupied by PKC-θ (μm^2^) versus the distance of the Golgi to the IS (μm) for eNOS and golgin-97–labeled G2A T cells. One hundred and ninety-one eNOS and 180 G2A cells were analyzed. (C) Fluorescence of PKC-θ (AU) at the IS of eNOS and G2A T cells. The mean ± standard deviation (SD) is shown. *** *p* ≤ 0.001. (D) Real-time confocal fluorescence microscopy of eNOS T cells transiently transfected with PKC-θ-monomeric red fluorescent protein (mRFP) and conjugated with SEB-pulsed Raji APCs (asterisk). Selected frames show the translocation of eNOS-GFP (green) and the area occupied by PKC-θ-mRFP (red) at the time indicated. Bar = 6 μm. *n* = 6. E) Area (μm^2^) occupied by PKC-θ-mRFP (●) and the distance (μm) of eNOS-GFP to the IS (○) in the T cell studied in (D) were represented versus the time from the establishment of the contact with the APC. Underlying data are provided in S1 Data.

To explore the role played by eNOS in the organization of signaling microclusters at the IS, we analyzed the localization of PKC-θ in conjugates between Raji B cells pulsed with the superantigen staphylococcal enterotoxin B (SEB) and TCR V_β_3–expressing CH7C17 Jurkat T cells, stably transfected with either eNOS-green fluorescent protein (GFP) (eNOS) or its inactive cytoplasmic G2A mutant (G2A) [30–32] (Fig 1). PKC-θ concentrated at the c-SMAC in 40%–50% of control CH7C17 and G2A T cells, whereas its coalescence to this area was reduced by half in eNOS T cells, a reduction also observed for CD28 (Fig 1A and S1 Fig). X–Z plane projections of the IS and their corresponding fluorescence profiles illustrate in more detail the scattered distribution of PKC-θ and CD28 at the IS of eNOS T cells (Fig 1A and S1 Fig). Moreover, the localization of PKC-θ at the c-SMAC of eNOS T cells was restored by the silencing of eNOS (S2A Fig), which reduced NO production to levels similar to those found in T lymphoblasts, parental, and eNOS T cells treated with the NOS inhibitor Nitro-L-Arg-methyl-ester (L-NAME) (S2B Fig). Furthermore, L-NAME increased the coalescence of PKC-θ at the c-SMAC of primary human T lymphoblasts (S2C Fig).

**Fig 2:**
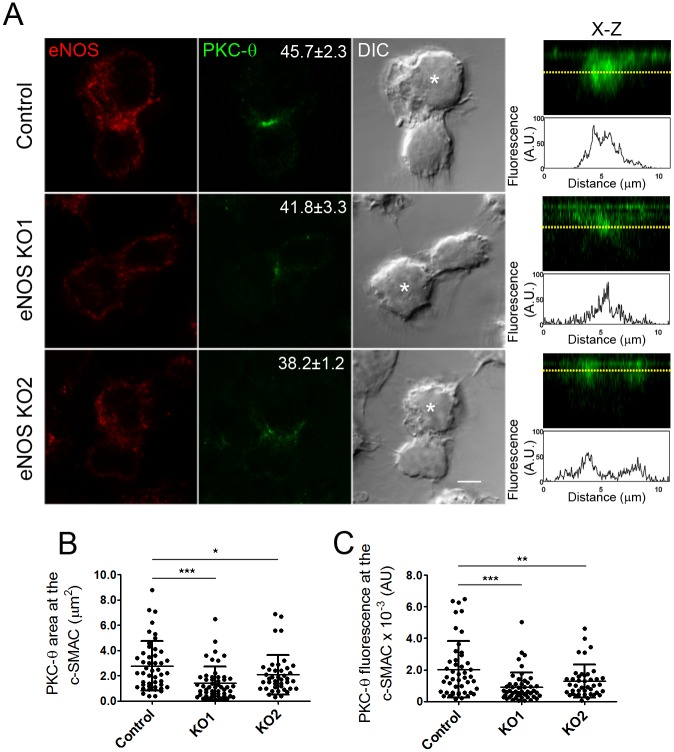
Endothelial Nitric Oxide Synthase (eNOS) deletion retains Protein Kinase C-θ (PKC-θ) at the central Supramolecular Activation Cluster (c-SMAC). (A) Localization of PKC-θ (green) and eNOS (red) in the immune synapse (IS) of control and eNOS KO1 and KO2 CH7C17 T cells, conjugated for 20 min with staphylococcal enterotoxin B (SEB)-pulsed Raji antigen-presenting cells (APCs) (asterisks). Bar = 4 μm. Percentages of cells with PKC-θ concentrated at the c-SMAC are indicated as mean ± standard error of the mean (SEM). *n* = 3. On the right, X–Z plane projections of PKC-θ at the IS and fluorescence profiles along the yellow dotted lines are shown. (B-C) Area (μm^2^) (B) and fluorescence of PKC-θ (AU) (C) at the c-SMAC of control and eNOS KO1 and KO2 CH7C17 T cells. Forty-seven control, 50 eNOS KO1, and 41 KO2 T cells were analyzed. * *p* ≤ 0.05, ** *p* ≤ 0.01, *** *p* ≤ 0.001. Underlying data are provided in S1 Data.

In an attempt to explore whether eNOS regulates the localization of PKC-θ from the IS, we analyzed the position of eNOS and the area and fluorescence of PKC-θ in SEB-specific T cell–APC conjugates. Data revealed that the distance of Golgi-associated eNOS to the IS inversely correlated with the area occupied by PKC-θ, a finding less easily observed in golgin-97–labeled G2A T cells (Fig 1B). The regulation exerted by eNOS on the localization of PKC-θ at the IS did not seem merely attributed to plasma membrane changes since the area occupied by the IS-independent T cell marker CD7 at the T cell–APC contact site did not differ among eNOS, G2A, and parental CH7C17 T cells (S3 Fig). Furthermore, fluorescence intensity from PKC-θ in the IS was higher in eNOS than in G2A T cells (Fig 1C). Those results were corroborated in live eNOS T cells transfected with PKC-θ-monomeric red fluorescent protein (mRFP) (Fig 1D and 1E). The action exerted by eNOS on the localization of PKC-θ was also assessed in CH7C17 T cells knocked-out for endogenous eNOS through a clustered regularly interspaced short palindromic repeats (CRISPR)/CRISPR-associated protein 9 (Cas9) system, in which NO production as response to TCR triggering is severely impaired (S4 Fig). Although no significant differences were found among control and two eNOS knockout (KO) CH7C17 T cell clones regarding the percentage of cells bearing PKC-θ concentrated at the c-SMAC, both the amounts and area of PKC-θ at the c-SMAC were considerably lower in eNOS KO than in control CH7C17 T cells (Fig 2A–2C). Collectively, these results strongly suggest that the recruitment and localization of PKC-θ at the IS is regulated by the compartmentalized activity of eNOS near the IS.

### eNOS partially colocalizes with F-actin at the IS and controls TCR-triggered actin polymerization

**Fig 3:**
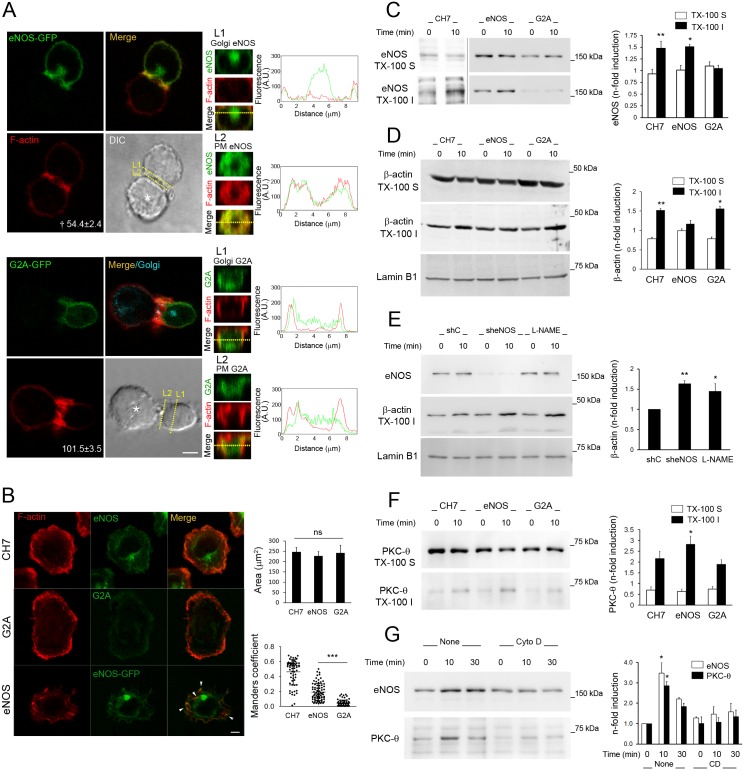
Endothelial Nitric Oxide Synthase (eNOS) associates with F-actin and reduces actin polymerization at the Immune Synapse (IS). (A) Localization of phalloidin-Alexa568–labeled F-actin (red) and G2A- or eNOS-green fluorescent protein (GFP) (green) in CH7C17 T cells conjugated with staphylococcal enterotoxin B (SEB)-pulsed Raji antigen-presenting cells (APCs) (asterisks). The Golgi of G2A T cells was labeled with golgin-97 (blue). Bar = 4 μm. The fluorescence of F-actin (AU) at the IS of eNOS and G2A T cells was represented as the mean ± standard error of the mean (SEM). † *p* ≤ 0.01. Eighty-four G2A and 100 eNOS T cells were analyzed. On the right, X–Z plane projections of F-actin and eNOS- or G2A-GFP and their corresponding fluorescence profiles along L1 (Golgi) and L2 (plasma membrane, PM) are shown. (B) Localization of F-actin (red) and eNOS, G2A-, or eNOS-GFP (green) in CH7C17 T cells stimulated on CD3 Ab-coated coverslips for 15 min. Arrow heads point to eNOS-GFP and F-actin colocalization. Bar = 4 μm. On the right, the cell spreading areas (μm^2^) and the Manders coefficients for colocalization between F-actin and eNOS or G2A are represented as mean ± SEM and ± standard deviation (SD), respectively. At least 60 cells were analyzed for each cell type. *** *p* ≤ 0.001. ns, not significant. (C-D) eNOS, eNOS-GFP and G2A-GFP (C), and β-actin (D) in Triton X-100 (TX-100)–soluble (TX-100 S) and–insoluble (TX-100 I) fractions from CH7C17, eNOS, and G2A T cells, stimulated for 10 min with cross-linked CD3 Ab. Lamin B1 is also shown. *n* = 4. In (C), the black line demarks immunoblots corresponding to separate analysis of endogenous and GFP-tagged eNOS. (E) β-actin in TX-100 I fractions from eNOS T cells treated with 300 μM Nitro-L-Arg-methyl-ester (L-NAME) or transduced with control or eNOS short hairpin RNAs (shRNAs) and stimulated as in (C-D). *n* = 4. (F) PKC-θ in TX-100 S and TX-100 I fractions from CH7C17, eNOS, and G2A T cells, stimulated for 10 min with cross-linked CD3 Ab. *n* = 3. (G) eNOS-GFP and PKC-θ in TX-100 I fractions from eNOS T cells, pretreated or not for 15 min with 1 μM Cytochalasin D and stimulated as in (C-D) for the time indicated. *n* = 3. On the right, the corresponding inductions of eNOS (C and G), β-actin (D-E), and PKC-θ (F-G) in TX-100 S and TX-100 I cell fractions are shown as mean ± SEM. * *p* ≤ 0.05, ** *p* ≤ 0.01. Underlying data are provided in S1 Data.

A direct interaction between eNOS and F-actin has been previously reported [33]. To study whether eNOS controls the localization of PKC-θ at the c-SMAC from the actin cytoskeleton, we analyzed F-actin in SEB-specific conjugates between Raji APCs and eNOS or G2A T cells. Plasma membrane–associated eNOS-GFP, but neither G2A-GFP nor Golgi-associated eNOS-GFP, codistributed with F-actin around the c-SMAC, and the levels of F-actin at the IS were lower in eNOS than in G2A T cells (Fig 3A). To avoid the interference of F-actin from APCs, the localization of eNOS and F-actin was also assessed in T cells settled on anti-CD3 Ab-coated coverslips (Fig 3B). No broad differences were found among the cell types studied regarding gross cell morphology and spreading (Fig 3B, upper right graph); however, whereas the lamellipodium of CH7C17 and G2A T cells showed a well-conformed F-actin–rich border, a less organized array of F-actin was observed in eNOS T cells (Fig 3B). Of note, endogenous eNOS and eNOS-GFP partially colocalized with F-actin (Fig 3B, lower right graph), suggesting that, upon TCR triggering, eNOS would be involved in the control of actin polymerization and lamellipodium organization. To test this, we analyzed the content of eNOS and β-actin in Triton X-100 (TX-100)–soluble and–insoluble/cytoskeletal fractions from CH7C17, G2A, and eNOS T cells. In steady-state conditions, G2A mainly associated with the soluble fraction. In contrast, endogenous eNOS and eNOS-GFP distributed between soluble and cytoskeletal fractions, increasing in the latter upon TCR-engagement (Fig 3C). Interestingly, β-actin increased in cytoskeletal fractions from CH7C17 and G2A T cells stimulated with CD3 Ab, reaching lower levels in eNOS T cells (Fig 3D)—an action dependent on eNOS-derived NO since both the interference of eNOS and its inhibition with L-NAME increased the levels of F-actin in CD3-stimulated eNOS T cells (Fig 3E).

We also assessed the presence of PKC-θ in TX-100–insoluble cell fractions. A small but significant amount of PKC-θ was recruited to the cytoskeleton in response to TCR engagement and this increased by overexpression of eNOS (Fig 3F), an effect depending on actin since the pretreatment of cells with the actin-depolymerizing agent cytochalasin D diminished the recruitment of both eNOS-GFP and PKC-θ to the cytoskeletal fraction (Fig 3G).

These results indicate that eNOS translocates to the actin cytoskeleton and controls actin polymerization in response to TCR engagement, which may regulate the recruitment of PKC-θ to the IS.

### eNOS controls F-actin clearance and the inward flow of actin and PKC-θ at the IS

**Fig 4:**
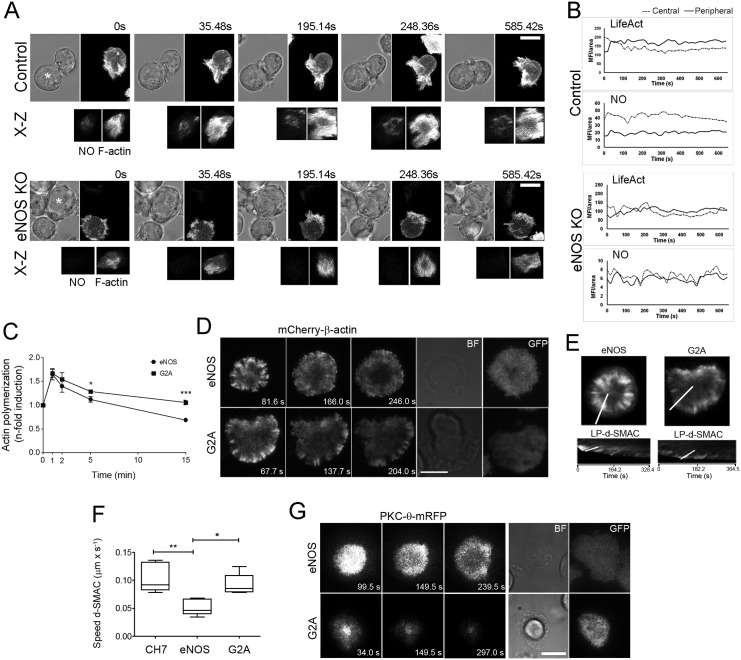
Endothelial Nitric Oxide Synthase (eNOS) regulation of F-actin and Protein Kinase C-θ (PKC-θ) dynamics in T cells. (A) Time-lapse confocal video microscopy imaging of live control and eNOS knockout (KO) CH7C17 T cells transfected with LifeAct- enhanced green fluorescent protein (EGFP) and conjugated with staphylococcal enterotoxin B (SEB)-pulsed Raji antigen-presenting cells (APCs) (asterisks). T cells were loaded with nitric oxide (NO) probe DAR-4M AM prior conjugation (30 min, 37°C, 0.5 μM). A maximal projection of the Z stack is shown for fluorescence. A bright field corresponds to a unique focus plane. Bar = 10 μm. Representative time-lapse experiments are shown. 3D reconstructions from the immune synapse (IS) are shown for NO and LifeAct-EGFP at different time points. See also S1 and S2 Movies. (B) Graphs showing the density of LifeAct-EGFP and NO fluorescence from the central and peripheral areas of 3D reconstructions of the IS (mean fluorescence intensity (MFI) per area) from the time lapses shown in (A). Control, *n* = 4; eNOS KO, *n* = 7. (C) Flow cytometry analysis of phalloidin-Alexa647–labeled F-actin in eNOS and G2A T cells, stimulated on CD3 Ab for the time indicated. F-actin induction was plotted as mean ± standard error of the mean (SEM). *n* = 3. * *p* ≤ 0.05, *** *p* ≤ 0.001. (D) Time-lapse total internal reflection fluorescence microscopy (TIRFM) of speckle mCherry-β-actin in eNOS and G2A T cells, stimulated on CD3 Ab-coated chambers. Bar = 10 μm. See also S3 Movie. (E) Kymographs and lamellipodium-like distal supramolecular activation cluster (d-SMAC) slope calculations of speckle mCherry-β-actin along the white line depicted on the projections of the movie frames from the eNOS and G2A T cells studied in (D). (F) Box and whisker plot of speckle mCherry-β-actin velocity in CH7C17, eNOS, and G2A T cells, analyzed as in (E). The medians are represented. *n* = 5. * *p* ≤ 0.05, ** *p* ≤ 0.01. (G) Time-lapse TIRFM of PKC-θ-monomeric red fluorescent protein (mRFP) in eNOS and G2A T cells stimulated on CD3 Ab-coated chambers. Bar = 10 μm. See also S4 and S5 Movies. *n* = 6. Underlying data are provided in S1 Data.

To explore whether eNOS-derived NO regulates the spatio-temporal organization of actin at the IS, LifeAct-enhanced GFP (EGFP)–transfected control and eNOS KO T cells were loaded with the NO probe DAR-4M AM, and the fluorescence of F-actin and the release of NO near the IS were simultaneously captured every 17.74 s by confocal fluorescence microscopy (Fig 4A and 4B). We found that, within the first 35 s from the establishment of initial cell contacts with APCs, both control and eNOS KO T cells similarly accumulate F-actin across the IS (Fig 4A and 4B, S1 and S2 Movies). However, whereas the F-actin levels at the central area of control T cells were progressively reduced within the next 1–3 min, the clearance of F-actin in eNOS KO T cells was considerably delayed, and after 5 min, F-actin progressively accumulated at the central area of eNOS KO but not control T cells, the latter showing a more apparent F-actin–rich ring-shaped d-SMAC (Fig 4A and 4B, S1 and S2 Movies). Regarding NO, its levels near the IS progressively increased from the establishment of initial cell contacts, reaching higher levels in control that in eNOS KO CH7C17 T cells (Fig 4A and 4B, S1 and S2 Movies).

The kinetics of actin polymerization in eNOS and G2A T cells were also investigated (Fig 4C–4F). Flow cytometry analysis of phalloidin-labeled F-actin in cells stimulated for 15 min on CD3 Ab-coated surfaces showed two well-defined phases: (i) a burst of actin polymerization that reached maximum values around the first minute of stimulation, followed by (ii), a more extended low rate of depolymerization that after 15 min returned F-actin to near basal levels (Fig 4C). Strikingly, whereas the burst of actin polymerization was similar in eNOS and G2A T cells, actin-depolymerization was accelerated in eNOS T cells, maintaining lower levels of F-actin relative to G2A T cells (Fig 4C). To visualize the spatio-temporal dynamics of β-actin in eNOS and G2A T cells, we transfected them with an mCherry-β-actin low copy expression plasmid [34] and performed time-lapse speckle total internal reflection fluorescence microscopy (TIRFM) upon activation on CD3 Ab-coated coverslips. Cells spread within the first minute, forming characteristic β-actin–rich lamellipodia that were more obvious and dynamic in G2A than in eNOS T cells (Fig 4D and S3 Movie). Next, by kymograph analyses, we compared the retrograde flow of mCherry-β-actin in the lamellipodium-like d-SMAC of TCR-engaged CH7C17, eNOS, and G2A T cells. β-actin moved inward slower in eNOS than in CH7C17 or G2A T cells (average rates of 0.052, 0.104, and 0.092 μm s^-1^, respectively) (Fig 4E and 4F). Additionally, tracking of PKC-θ-mRFP in cells stimulated on CD3 Ab showed that microclusters of PKC-θ concentrated at a central area in G2A T cells but distributed peripherally in eNOS T cells (Fig 4G, S4 and S5 Movies).

Overall, these results indicate that eNOS controls the actin cytoskeleton at the IS, fostering early and sustained F-actin clearance at the central area but reducing the centripetal retrograde flow of actin and the inward movement of PKC-θ microclusters from the lamellipodium-like d-SMAC.

### eNOS increases the activation of PKC-θ in T cells

**Fig 5:**
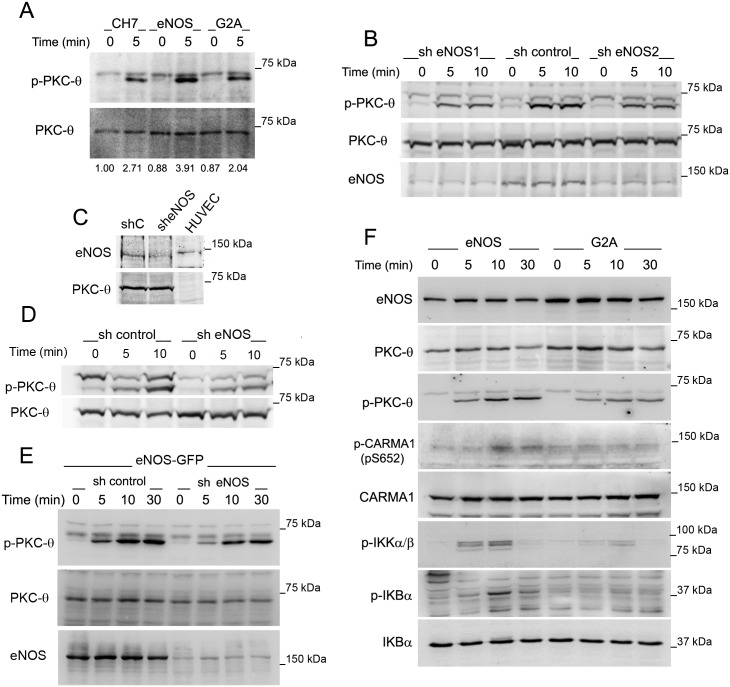
Endothelial Nitric Oxide Synthase (eNOS) positively regulates Protein Kinase C-θ (PKC-θ) activation. (A) PKC-θ Thr538 phosphorylation in CH7C17, eNOS, and G2A T cells, stimulated for 10 min with cross-linked CD3 Ab. On the bottom, densitometric quantification of PKC-θ phosphorylation is shown as the mean fold induction of three independent experiments. Underlying data are provided in S1 Data. (B) PKC-θ phosphorylation in parental CH7C17 T cells, transduced with control or two independent eNOS short hairpin RNAs (shRNAs) (sh eNOS1 and 2) and stimulated 72 h later as in (A) for the time indicated. *n* = 2. (C) eNOS expression in primary human T lymphoblasts from staphylococcal enterotoxin E (SEE)-stimulated peripheral blood lymphocytes (PBLs), transduced for 72 h with either control (shC) or eNOS (sheNOS) shRNAs. Human umbilical vein endothelial cells (HUVEC) extracts were loaded as control. (D) PKC-θ phosphorylation in human T lymphoblasts interfered for eNOS as in (C) and subsequently stimulated with SEE-pulsed Raji antigen-presenting cells (APCs) (cell ratio = 4:1) for the time indicated. *n* = 3. (E) PKC-θ phosphorylation in eNOS T cells, transduced with control or eNOS shRNAs and stimulated 72 h later with SEB-pulsed Raji APCs for the time indicated. *n* = 4. (F) eNOS and G2A T cells were stimulated with CD3 Ab for the time indicated. eNOS, phospho-IKKα/β, and total and phosphorylated PKC-θ, CARMA1, and IκBα were detected by immunoblot. *n* = 3.

Microcluster signaling is generated and sustained at the lamellipodium-like d-SMAC [12,35]. The persistence of PKC-θ in the peripheral region of eNOS-overexpressing T cells led us to examine its activation state. Phosphorylation of PKC-θ on Thr538, as a response to stimulation with CD3 Ab, was higher in eNOS than in either G2A or CH7C17 T cells (Fig 5A). The specific action of eNOS on the phosphorylation of PKC-θ was corroborated by its knockdown, which broadly reduced endogenous expression of eNOS and TCR-triggered phosphorylation of PKC-θ in parental CH7C17 T cells and primary human T lymphoblasts (Fig 5B–5D). Similarly, upon stimulation with SEB-pulsed APCs, knockdown of eNOS reduced PKC-θ phosphorylation in eNOS T cells but not in G2A T cells (Fig 5E and S5 Fig).

To assess the functional impact of the regulation of PKC-θ by eNOS, we analyzed the NF-κB pathway in eNOS and G2A T cells stimulated with CD3 Ab (Fig 5F), focusing on CARMA1, IκB, and IκB kinase (IKK), as well known components of the TCR-triggered cascade of NF-kB activation [36]. Phosphorylation of CARMA1 on Ser652, a PKC-θ target equivalent to Ser657 in mouse cells [37], IKKα/β, and IκBα were enhanced in eNOS with respect to G2A T cells (Fig 5F).

These results indicate that eNOS positively regulates the activation of PKC-θ at the IS.

### eNOS-derived NO regulates PKC-θ localization and activation through a cGMP-independent mechanism

**Fig 6:**
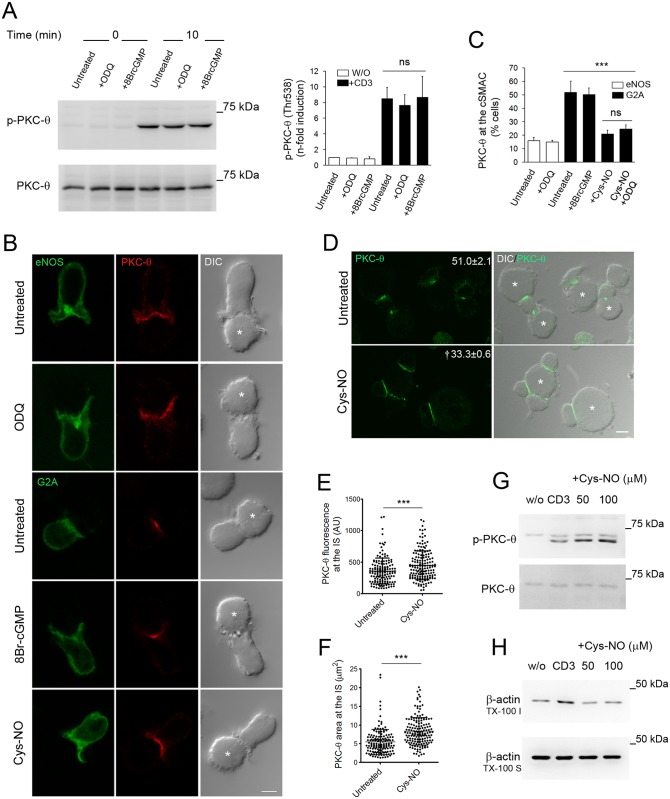
Endothelial Nitric Oxide Synthase (eNOS) regulates Protein Kinase C-θ (PKC-θ) through a cyclic Guanosine Monophosphate (cGMP)-independent mechanism. (A) PKC-θ Thr538 phosphorylation in eNOS T cells, stimulated with cross-linked CD3 Ab for 10 min either after 30 min pretreatment with 3 μM 1H-[1,2,4]oxadiazolo[4,3-a]quinoxalin-1-one (ODQ) or in the presence of 100 μM 8-bromoguanosine 3',5'-cyclic monophosphate (8Br-cGMP). On the right, the induction of PKC-θ phosphorylation was represented for each condition as the mean ± standard error of the mean (SEM). *n* = 3. ns, not significant. (B) Localization of PKC-θ (red) and eNOS- or G2A-GFP (green) in eNOS and G2A T cells, conjugated for 20 min with staphylococcal enterotoxin B (SEB)-pulsed Raji antigen-presenting cells (APCs) (asterisks). Where indicated, cells were treated with 3 μM ODQ, 100 μM 8Br-cGMP, or 50 μM S-nitroso-L-cysteine (Cys-NO). Bar = 4 μm. (C) Quantitative analysis of the localization of PKC-θ at the central supramolecular activation cluster (c-SMAC) of T cells, studied as in (B). Data are mean ± SEM. *n* = 3. At least 180 T cell–APC conjugates were scored for each cell type and condition. ns, not significant, *** *p* ≤ 0.001. (D) Localization of PKC-θ in staphylococcal enterotoxin E (SEE)-specific primary human T lymphoblasts, conjugated for 20 min with SEE-pulsed Raji APCs (asterisks) ± 50 μM Cys-NO. Bar = 6 μm. Percentages of cells with PKC-θ concentrated at the c-SMAC are shown as mean ± SEM. *n* = 3. † *p* ≤ 0.01. (E-F) Fluorescence (AU) (E) and area (μm^2^) (F) of PKC-θ at the immune synapse (IS) of cells studied in (D) are shown as mean ± standard deviation (SD). One hundred and fifty-two untreated and 165 Cys-NO–treated cells were analyzed. *** *p* ≤ 0.001. (G) PKC-θ Thr538 phosphorylation in primary human T lymphoblasts, stimulated with cross-linked CD3 Ab ± Cys-NO. *n* = 3. (H) β-actin in TX-100–soluble (TX-100 S) and–insoluble (TX-100 I) fractions from primary human T lymphoblasts, stimulated as in (G). *n* = 3. Underlying data are provided in S1 Data.

The above results prompted us to explore the mechanisms by which eNOS controls the localization and activation of PKC-θ at the c-SMAC, focusing on soluble guanylate cyclase (sGC) as one of the most sensitive NO targets in cells. Neither 1H-[1,2,4]oxadiazolo[4,3-a]quinoxalin-1-one (ODQ), a potent inhibitor of sGC, nor 8-bromoguanosine 3',5'-cyclic monophosphate (8Br-cGMP), a cGMP analogue, exerted any significant effect on the activation of PKC-θ in eNOS T cells stimulated with CD3 Abs (Fig 6A). Furthermore, neither the dispersed distribution of PKC-θ at the IS of eNOS T cells nor its concentrated localization at the c-SMAC of G2A T cells were significantly altered by ODQ and 8Br-cGMP, respectively (Fig 6B and 6C). On the contrary, treatment of G2A T cells with Cys-NO, an NO donor that bears transnitrosylating activity, disturbed the localization of PKC-θ at the IS, irrespective of whether cells were pretreated with ODQ (Fig 6B and 6C). These results were validated in primary human T lymphoblasts. Cys-NO treatment increased the fluorescence from PKC-θ at the IS but impaired its coalescence to the c-SMAC of superantigen staphylococcal enterotoxin E (SEE)-specific T cell–APC conjugates (Fig 6D–6F), and increased PKC-θ phosphorylation but decreased F-actin levels upon CD3 stimulation (Fig 6G and 6H).

These findings indicate that eNOS-derived NO regulates the activation and organization of PKC-θ at the IS via a cGMP-independent mechanism that may involve protein S-nitrosylation.

### eNOS stimulates β-actin S-nitrosylation on Cys374 upon TCR-triggering

**Fig 7:**
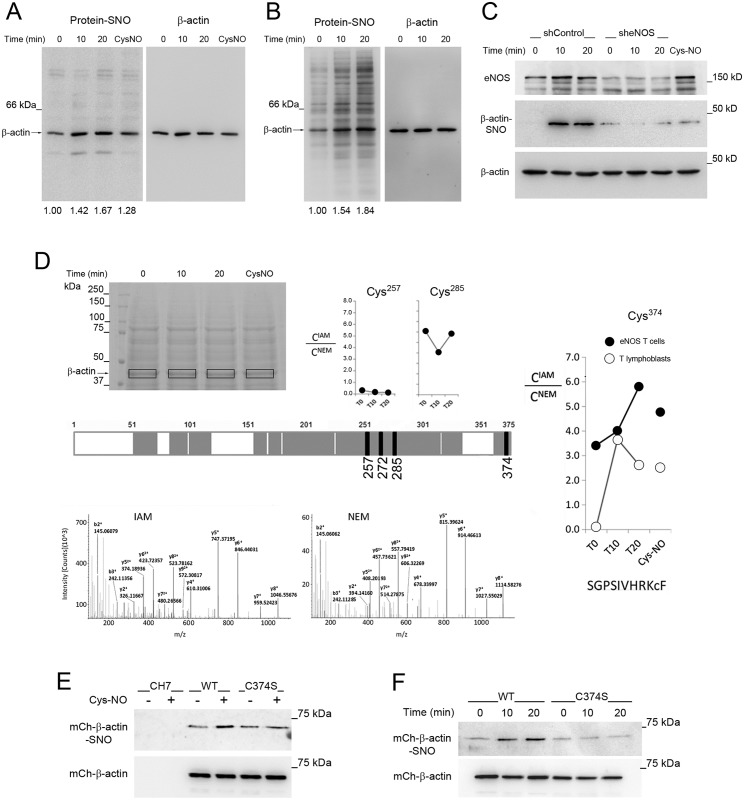
Endothelial Nitric Oxide Synthase (eNOS) S-nitrosylates β-actin on Cys374. (A-B) Biotin-switch analysis of β-actin S-nitrosylation in primary human T lymphoblasts (A) and eNOS T cells (B), stimulated for the time indicated with CD3 Ab ± 50 μM S-nitroso-L-cysteine (Cys-NO) and staphylococcal enterotoxin B (SEB)-pulsed Raji antigen-presenting cells (APCs), respectively. Quantitative analysis of β-actin S-nitrosylation is indicated at the bottom. *n* = 3 (A) and *n* = 5 (B). (C) Biotin-switch analysis of β-actin S-nitrosylation in eNOS T cells, transduced with control or eNOS shRNAs and subsequently stimulated with SEB-pulsed Raji APCs for the time indicated. As control, cells were treated with 50 μM Cys-NO. *n* = 3. (D) Proteomic analysis of β-actin S-nitrosylation. SDS-PAGE Coomassie staining of protein samples from cells treated as in (A-B), of which β-actin–containing gel pieces were excised for proteomic studies (upper left). The schematic representation of β-actin shows: GluC-digested peptide matching for protein identification (grey), non-covered protein sequence (white areas), and the four characterized Cys of the C-terminal region (black lines). In the upper part, the intensities of the C^IAM^/C^NEM^ ratio for β-actin–derived peptides containing Cys257 and 285 are represented. On the right, the ratios for the Cys374-containing peptide spanning the sequence SGPSIVHRKCF are shown for eNOS T cells and primary human T lymphoblasts; below, the fragmentation spectra for both adducts showing the y-fragmentation series are depicted. *n* = 2. (E) mCherry-β-actin S-nitrosylation in CH7C17, wild-type (WT), and C374S T cells treated with 50 μM Cys-NO. *n* = 3. (F) mCherry-β-actin S-nitrosylation in eNOS T cells transiently transfected with WT- or C374S-mCherry-β-actin and stimulated 24 h later with SEB-pulsed Raji APCs for the time indicated. *n* = 3. Underlying data are provided in S1 Data.

β-actin is among the major S-nitrosylated proteins detected in cells stimulated with NO [38]. To investigate whether eNOS S-nitrosylates β-actin in T cells, we performed biotin-switch assays, which consist of the specific biotinylation of proteins bearing S-nitrosylated Cys after the irreversible blockage of non-nitrosylated Cys. S-nitrosylation of β-actin increased in primary human T lymphoblasts stimulated with CD3 Ab (Fig 7A) and also in eNOS T cells stimulated with SEB-pulsed Raji APCs (Fig 7B), in which S-nitrosylation of β-actin was greatly reduced by knockdown of eNOS (Fig 7C).

Four Cys nearest to the C-terminus of β-actin: Cys 257, 272, 285, and 374, have been identified as targets of hyperoxia-induced S-nitrosylation in neutrophils [39]. To study whether one or more of these Cys were S-nitrosylated in T cells as a response to TCR triggering, we performed proteomic studies (Fig 7D). Cell extracts from eNOS T cells stimulated with SEB-pulsed Raji APCs were subjected to a nitrosothiol-specific assay similar to the biotin switch, with N-ethyl-maleimide (NEM) blocking agent but using the alkylating agent iodoacetamide (IAM) instead of HPDP-biotin to improve the fragmentation quality in mass spectrometry data from β-actin–derived peptides. We found that Cys257 was very poorly S-nitrosylated, and S-nitrosylation of Cys285 was higher, but changed slightly upon stimulation (Fig 7D). Regarding Cys272, the presence of Met in the peptide distributed the ion intensity in several adducts, impeding a quantitative estimation of its S-nitrosylation. Interestingly, S-nitrosylation of Cys374 strikingly increased upon TCR engagement, reaching levels comparable to those observed upon Cys-NO treatment (Fig 7D). Similarly, S-nitrosylation on Cys374 increased in primary human T lymphoblasts stimulated with CD3 Ab, remaining below the S-nitrosylation levels observed in eNOS T cells (Fig 7D). Furthermore, biotin-switch analysis of CH7C17 and eNOS T cells expressing wild-type (WT)- or C374S-mCherry-β-actin and treated with Cys-NO or SEB-pulsed Raji APCs showed that S-nitrosylation of WT β-actin increased with respect to the C374S mutant (Fig 7E and 7F), indicating that, even if basal S-nitrosylation of other Cys could be observed, as a response to TCR engagement eNOS-derived NO mainly S-nitrosylated β-actin on Cys374.

### β-actin S-nitrosylation on Cys374 impairs actin binding to profilin-1 (PFN1)

**Fig 8:**
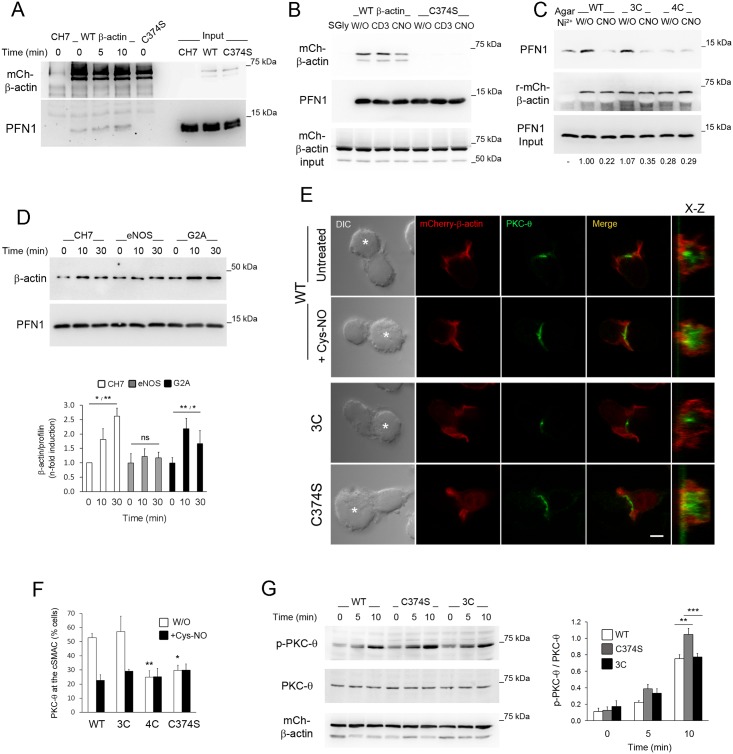
β-actin S-nitrosylation on Cys374 impairs actin binding to profilin-1 (PFN1) and regulates localization and activation of Protein Kinase C-θ (PKC-θ) at the Immune Synapse (IS). (A) Coprecipitation of PFN1 with mCherry-β-actin from wild-type (WT)- and C374S-mCherry-β-actin–expressing T cells activated with staphylococcal enterotoxin B (SEB)-pulsed Raji antigen-presenting cells (APCs) for the time indicated. Immunoprecipitation from parental CH7C17 cells were used as negative control. The corresponding mCherry-β-actin and PFN1 inputs are shown. *n* = 3. (B) Binding of WT- and C374S-mCherry-β-actin to PFN1 precipitated with poly-L-Pro-coupled Sepharose from WT and C374S T cells, stimulated for 10 min with cross-linked CD3 Ab ±100 μM S-nitroso-L-cysteine (Cys-NO). Gly-coupled Sepharose was used as control (SGly). *n* = 3. (C) Binding of PFN1 from CH7C17 T cell extracts to His-tagged WT-, 3C-, and 4C-mCherry-β-actin coupled to agarose-Ni^2+^ and pretreated/S-nitrosylated or not with 100 μM Cys-NO. Agarose-Ni^2+^ was used as control (AgarNi^2+^). On the bottom, the corresponding densitometric quantification of PFN1 binding to recombinant actins is shown as the mean fold induction from three independent experiments. (D) β-actin binding to PFN1 precipitated with poly-L-Pro-coupled Sepharose from CH7C17, eNOS, and G2A T cells, stimulated with CD3 Ab for the time indicated. *n* = 7. Normalized inductions of profilin-actin complexes are shown as mean ± standard error of the mean (SEM). ns, not significant, * *p* ≤ 0.05, ** *p* ≤ 0.01. (E) Localization of PKC-θ (green) and WT-, C374S-, or 3C-mCherry-β-actin (red) in CH7C17 T cells, conjugated for 20 min with SEB-pulsed Raji APCs (asterisks). Where indicated, WT-mCherry-β-actin T cells were treated with 50 μM Cys-NO. On the right, X–Z plane projections of mCherry-β-actin and PKC-θ at the IS are depicted. Bar = 4 μm. (F) Percentages of cells with PKC-θ concentrated at the central supramolecular activation cluster (c-SMAC) for WT-mCherry-β-actin and the three Cys-to-Ser mutants treated or not with Cys-NO. The mean ± SEM is depicted. *n* = 3. At least 100 T cell–APC conjugates were scored for each cell type and condition, * *p* ≤ 0.05, ** *p* ≤ 0.01. (G) Time-course analysis of PKC-θ Thr538 phosphorylation in WT, 3C, and C374S T cells, stimulated with SEB-pulsed Raji APCs. The histogram shows the normalized phosphorylation of PKC-θ as mean ± SEM. *n* = 4. ** *p* ≤ 0.01, *** *p* ≤ 0.001. Underlying data are provided in S1 Data.

Mutation to Ser on the Cys374 of actin interferes with formation of PFN1-actin complexes in vitro [40]. Before studying whether S-nitrosylation on Cys374 also impaired the binding of β-actin to PFN1, we confirmed by coprecipitation experiments that PFN1 did not bind the C374S β-actin mutant in cells (Fig 8A). In contrast, PFN1 binding to wild-type mCherry-β-actin was detected in nonstimulated CH7C17 T cells and increased upon stimulation with SEB-pulsed Raji APCs (Fig 8A). An important feature of profilin is its capability to bind actin-associated and scaffolding proteins through their poly-L-Pro regions [41]. We took advantage of that to perform pull-down experiments with poly-L-Pro-coated sepharose beads, analyzing the interaction of PFN1 with β-actin in the presence or absence of Cys-NO (Fig 8B). Complexes between PFN1 and WT-mCherry-β-actin increased in T cells upon CD3 stimulation and diminished by Cys-NO treatment, which partially reproduced the defective interaction of C374S β-actin with PFN1 (Fig 8B). To corroborate that S-nitrosylation on Cys374 regulates the interaction of β-actin with PFN1, His-tagged recombinant WT mCherry-β-actin or its corresponding Cys-to-Ser mutants: 3C, on C257, 272, and 285; and 4C, on C257, 272, 285, and 374, whose sole difference was the mutation to Ser of Cys374 in 4C but not in 3C β-actin (S6A Fig), were in vitro translated and folded in the presence of the eukaryotic chaperonin CCT/TRiC (S6B–S6F Fig), in a similar way as described previously [42]. Translated actins were bound to agarose-Ni^2+^ beads, S-nitrosylated in vitro with Cys-NO, and allowed to bind PFN1 from CH7C17 T cell extracts by pull-down assays (Fig 8C). Whereas PFN1 barely associated with 4C β-actin, it bound considerably to WT- and 3C β-actin, decreasing significantly by pretreatment with Cys-NO (Fig 8C). Furthermore, stimulation via CD3 increased the formation of profilin-actin complexes in CH7C17 and G2A but not in eNOS T cells (Fig 8D), strongly suggesting that eNOS-mediated S-nitrosylation on Cys374 regulates the binding of β-actin to PFN1.

### β-actin S-nitrosylation on Cys374 controls PKC-θ localization and activation at the IS by impairing actin binding to PFN1

To study whether S-nitrosylation of β-actin on Cys374 regulated the localization and activation of PKC-θ by impairing actin binding to PFN1, CH7C17 T cells stably expressing comparable levels of WT-mCherry-β-actin or its corresponding Cys-to-Ser mutants 3C, 4C, and C374S (S7A Fig) were analyzed by confocal fluorescence microscopy. PKC-θ concentrated at the c-SMAC of WT- and 3C T cells and was similarly dispersed upon Cys-NO treatment (Fig 8E and 8F, S7B Fig). Interestingly, PKC-θ dispersed at the IS of cells expressing the mutant 4C or C374S, even in the absence of Cys-NO (Fig 8E and 8F, S7B Fig), as CD28 did on the IS of C374S mCherry-β-actin–expressing T cells (S8 Fig); furthermore, phosphorylation of PKC-θ increased in C374S T cells with respect to WT- and 3C T cells stimulated with SEB-pulsed Raji APCs (Fig 8G), indicating that the S-nitrosylation–defective C374S mutant mimics the actions exerted by the nitrosylating activity of eNOS-derived NO and Cys-NO on the localization and activation of PKC-θ.

**Fig 9:**
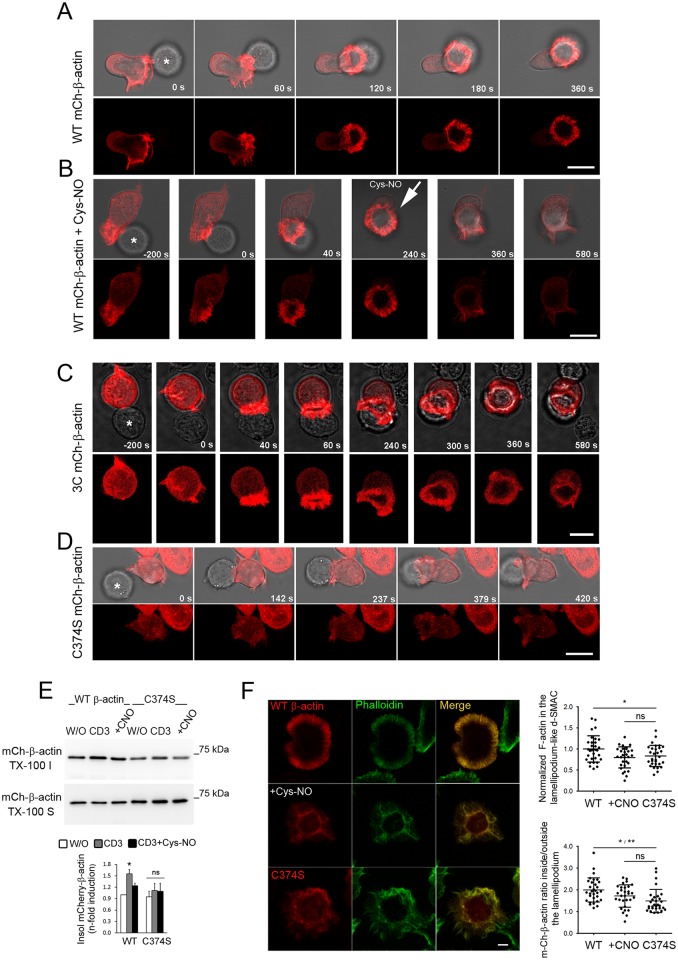
β-actin S-nitrosylation on Cys374 reduced actin polymerization at the lamellipodium-like distal Supramolecular Activation Cluster (d-SMAC). (A-D) Time-lapse confocal fluorescence microscopy of wild-type (WT)- (A-B), 3C- (C), and C374S-mCherry-β-actin (D) in CH7C17 T cells conjugated with staphylococcal enterotoxin B (SEB)-pulsed Raji antigen-presenting cells (APCs) (asterisks). In (B), 50 μM S-nitroso-L-cysteine (Cys-NO) was added 240 s after the establishment of the T cell–APC conjugate (arrow). For each cell type and condition, a representative experiment out of at least five is shown. Bar = 10 μm. See also S6, S7, S8 and S9 Movies. (E) mCherry-β-actin in TX-100–insoluble/F-actin (TX-100 I) and–soluble/G-actin (TX-100 S) fractions from WT and C374S T cells, stimulated for 10 min with cross-linked CD3 Ab ±100 μM Cys-NO (CNO). Inductions of mCherry-β-actin in TX-100–insoluble/F-actin cell fractions are shown as mean ± standard error of the mean (SEM). *n* = 5. * *p* ≤ 0.05. ns, not significant. (F) Localization of phalloidin-Alexa488–labeled F-actin (green) and WT- or C374S-mCherry-β-actin (red) in CH7C17 T cells stimulated on CD3 Ab-coated coverslips for 15 min. Bar = 4 μm. The upper right chart shows the normalized values of phalloidin-labeled F-actin in the lamellipodium-like d-SMAC of WT- and C374S-mCherry-β-actin T cells with respect to F-actin from WT-mCherry-β-actin T cells. On the lower right chart, values corresponding to lamellipodium-like d-SMAC inside/outside ratios for WT- and C374S-mCherry-β-actin are shown. Where indicated, WT mCherry-β-actin T cells were treated with 50 μM Cys-NO. The mean ± standard deviation (SD) of at least 30 cells analyzed for each cell type and condition is shown. *p ≤ 0.05, **p ≤ 0.01. Underlying data are provided in S1 Data.

We also studied the impact of S-nitrosylation on Cys374 on the organization of β-actin in the IS (Fig 9A–9D). WT-mCherry-β-actin localized at the front of motile T cells in F-actin–rich filopodia and microspikes and, after contact with antigen-pulsed APCs, those completely reorganized to form the d-SMAC as a lamellipodium-like structure packed with parallel distributed F-actin bundles (Fig 9A and S6 Movie). Remarkably, most of the radially-arranged WT-mCherry-β-actin–containing F-actin bundles in the d-SMAC were disorganized by the addition of 50 μM Cys-NO (Fig 9B and S7 Movie). Interestingly, whereas the mutant 3C organized in bundles of F-actin at the lamellipodium-like d-SMAC, the C374S-mCherry-β-actin mutant did not (Fig 9C and 9D, S8 and S9 Movies). To explore whether β-actin S-nitrosylation on Cys374 may regulate the localization and activation of PKC-θ by reducing actin polymerization, fractionation of TX-100 extracts from WT- and C374S T cells stimulated with CD3 Ab and treated or not with Cys-NO was performed (Fig 9E). As a response to TCR engagement WT-mCherry-β-actin increased in the F-actin cell fraction, an effect prevented by treatment with Cys-NO (Fig 9E). Remarkably, recruitment of C374S-mCherry-β-actin to the F-actin fraction was impaired and remained almost unaltered after treatment with Cys-NO (Fig 9E). Moreover, WT-mCherry-β-actin incorporated into regularly spaced F-actin bundles at the lamellipodium-like d-SMAC of cells activated on CD3 Ab-coated coverslips, and those were disorganized by treatment with Cys-NO, increasing lamellar and cytoplasmic actin—a pattern of actin also observed in cells overexpressing the C374S β-actin mutant (Fig 9F), suggesting that S-nitrosylation on Cys374 regulates PKC-θ at the IS through rearrangement of the actin cytoskeleton.

**Fig 10:**
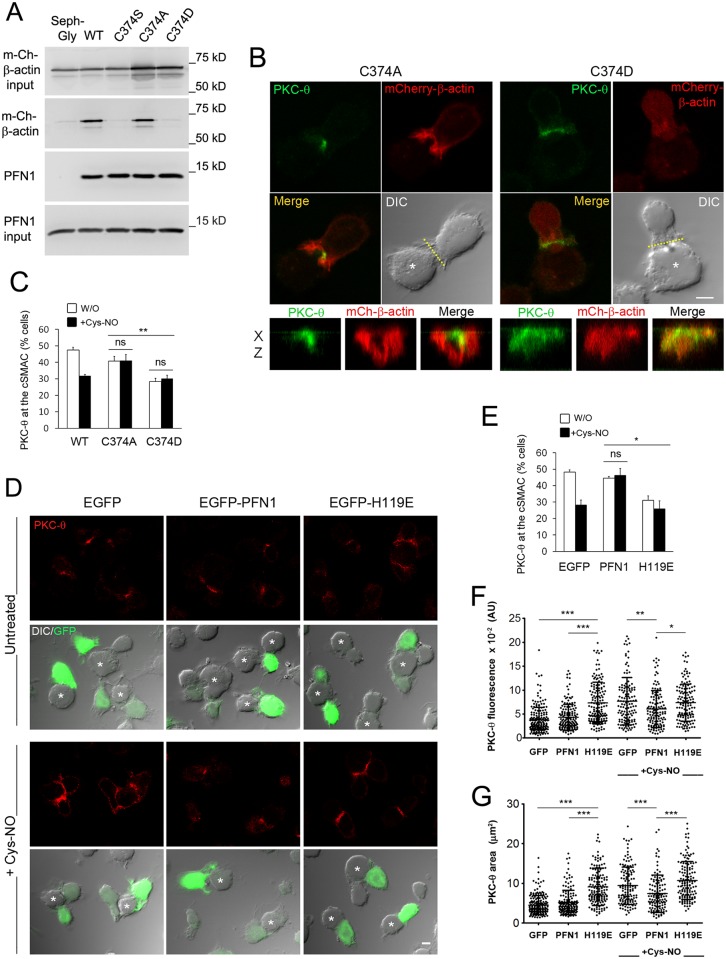
β-actin S-nitrosylation on Cys374 regulates Protein Kinase C-θ (PKC-θ) at the Immune Synapse (IS) by impairing actin binding to profilin-1 (PFN1). (A) Binding of WT-, C374S-, C374A-, and C374D-mCherry-β-actin to PFN1 precipitated with poly-L-Pro-coupled Sepharose from their respective CH7C17 T cell transfectants. Gly-coupled Sepharose was used as control (Seph-Gly). *n* = 2. (B) Localization of PKC-θ (green) and C374A- or C374D-mCherry-β-actin (red) at the IS of T cells conjugated with staphylococcal enterotoxin B (SEB)-pulsed Raji antigen-presenting cells (APCs) (asterisks). Bar = 4 μm. Below, X–Z projections of PKC-θ and mCherry-β-actin at the IS are shown. (C) Quantitative analysis of the localization of PKC-θ at the c-SMAC of WT-, C374A-, and C374D-mCherry-β-actin T cells treated or not with 50 μM S-nitroso-L-cysteine (Cys-NO). Percentages of cells showing PKC-θ concentrated at the central supramolecular activation cluster (c-SMAC) are indicated as mean ± standard error of the mean (SEM). *n* = 3. At least 127 cells were scored for each cell type and condition. ** *p* ≤ 0.01. (D) Localization of PKC-θ (red) at the IS of CH7C17 T cells transfected with EGFP, EGFP-PFN1, or -H119E (green) and conjugated with SEB-pulsed Raji APCs (asterisks) ±50 μM Cys-NO. Bar = 4 μm. (E-G) Quantitative analysis of the localization of PKC-θ at the c-SMAC of the cell types studied in (D). Percentages of cells with PKC-θ concentrated at the c-SMAC (E) and the fluorescence (F) and area (G) of PKC-θ at the IS are indicated as mean ± SEM (E) and ± standard deviation (SD) (F-G). *n* = 3. At least 133 cells were scored for each cell type and condition. * *p* ≤ 0.05, ** *p* ≤ 0.01, *** *p* ≤ 0.001. Underlying data are provided in S1 Data.

To corroborate that the reduced binding of PFN1 to β-actin S-nitrosylated on Cys374 is important for the actin rearrangement associated with the localization of PKC-θ at the IS, we generated CH7C17 T cells stably expressing mCherry-tagged constructs of the previously characterized C374A- and C374D-β-actin mutants [43] (S9A Fig). Similar to C374S, C374D β-actin neither organized in F-actin bundles nor bound to PFN1 (Fig 10A and 10B and S9B Fig). In contrast, C374A organized in F-actin and bound to PFN1 in a manner similar to WT β-actin (Fig 10A and 10B and S9B Fig). Interestingly, PKC-θ concentrated at the IS of C374A T cells and was barely disturbed by Cys-NO, whereas it was evenly dispersed in C374D T cells (Fig 10B and 10C), supporting the hypothesis that the binding of PFN1 to β-actin is important for the localization of PKC-θ at the IS. To further validate this, parental CH7C17 T cells were transfected with control EGFP, EGFP-PFN1 WT, or the actin binding-defective PFN1 mutant H119E [44]. Overexpression of H119E increased the recruitment and area of PKC-θ at the IS, disturbing its coalescence to the c-SMAC, whereas WT PFN1 but not H119E counteracted the actions exerted by Cys-NO on the recruitment and localization of PKC-θ (Fig 10D–10G).

Collectively, these results strongly suggested that in antigen-specific T cell–APC conjugates eNOS-derived NO S-nitrosylates β-actin on Cys374, a posttranslational modification that, by altering the formation of profilin-actin complexes, controls TCR-triggered actin polymerization and retrograde flow at the IS, regulating from the lamellipodium-like d-SMAC the localization and activation of PKC-θ at the c-SMAC.

## Discussion

Actin depolymerizing and stabilizing agents and the functional interference of the actin nucleator complex Arp2/3 disturb the architecture of the IS [13,24,25]; nevertheless, the mechanisms involved are still not fully understood, and the participation of additional factors has been proposed [26]. In this work we show that one of these factors is NO. Previous evidence indicates that NO rearranges the actin cytoskeleton in T lymphocytes and disturbs the organization of CD3 at the c-SMAC [8,45]. We have investigated herein the mechanisms underlying the impact of NO on the organization of the IS, focusing on PKC-θ as a c-SMAC hallmark whose activation depends on its proximity to the p-SMAC [17].

Our results show that, upon TCR triggering, eNOS translocates with the Golgi to the IS, interacting with F-actin and reducing actin polymerization. Although this interaction may be indirect, our findings are in agreement with previous studies in endothelial cells, which showed that eNOS interacts with cortical F-actin at the plasma membrane and with G-actin on the Golgi complex, activating the enzyme to produce NO [46]. Besides the regulation of the production of NO by β-actin, a complementary process has also been demonstrated through studies carried out in leukocytes, in which exposure to NO led to the remodeling of the actin cytoskeleton [45,47]. Our findings are thus consistent with the association of eNOS with F-actin early during the burst of actin polymerization induced by TCR triggering and thereafter with the depolymerization of β-actin close to the IS by local production of eNOS-derived NO.

Experimental models of T cell activation on surfaces coated with CD3 Ab have helped to establish that the lamellipodium plays a critical role in the organization and activation of signaling microclusters in the IS [35,48]. The lamellipodium shares structural and functional analogies with the d-SMAC, both showing similar patterns of F-actin organization and displaying extensive actin-based retrograde flow [12,49,50]. High-resolution 4D imaging studies of conjugates between cytotoxic T lymphocytes (CTLs) and target cells have recently confirmed and extended this issue by showing that polymerization and retrograde flow of actin increased in the periphery of the IS whereas it decreased in its central area [51]. Accordingly, our data show that eNOS partially colocalizes with F-actin at the lamellipodium-like d-SMAC, reducing actin polymerization and retrograde flow. Through this, eNOS-derived NO can facilitate sustained signaling by reducing from the d-SMAC the inward transit of signaling molecules associated with actin, as indicated by our TIRFM experiments in which transfection of eNOS, but not its G2A-defective mutant, retained PKC-θ out of the c-SMAC. However, besides the lateral movement of signaling microclusters, Golgi and centrosome translocation to and vesicle fusion at the IS (regulated by early actin depletion and the formation of soluble NSF attachment protein receptor [SNARE] complexes, respectively) are also important suppliers of signaling molecules to the c-SMAC [51,52]. In this regard, our complementary loss-of-function studies provide compelling evidence that eNOS favors early actin depletion and accumulation of PKC-θ at the IS; whether this may result through the regulation of vesicular traffic from the Golgi apparatus will need further research.

Although CD28 is not essential for the recruitment of PKC-θ to the IS, the coalescence of PKC-θ to the c-SMAC is promoted by CD28—requiring its cytoplamic tail and binding to CD80 on the APC [17,53]—and the association of PKC-θ with the actin cytoskeleton via filamin A, a process positively regulated by PKC-θ sumoylation, and by the actin-uncapping protein Rltpr, whose L432P mutant disturbs the localization of PKC-θ but not CD28 in the IS [21,54]; accordingly, we provide evidence that upon TCR engagement, eNOS fosters the recruitment of a fraction of PKC-θ to the actin cytoskeleton. Moreover, eNOS similarly controls the coalescence of PKC-θ and CD28 to the IS, suggesting that an Rltpr-independent mechanism may be involved. It is feasible thus that, as part of this mechanism, eNOS-derived NO interfered with the linking of the CD28–filamin A–PKC-θ complex to actin and its flow towards the c-SMAC such as it has been recently described for the desumoylation of PKC-θ, which impairs the coalescence of both PKC-θ and CD28 [21]. Whether the sumoylation of PKC-θ would be also involved in the actin-mediated mechanism by which NO regulates the localization and activation of PKC-θ at the IS remains a standing question.

Our data also show that eNOS-synthesized NO controls the localization and activation of PKC-θ at the c-SMAC through a cGMP-independent mechanism that involves S-nitrosylation of β-actin on Cys374. Because of the accessibility of Cys374 in the flexible C-terminal region of β-actin, its sulfhydryl group is highly reactive [55]. Specific labelling of Cys374 with the fluorescent probe TMR-5-maleimide renders actin non-polymerizable [56]. Further, S-glutathionylation on Cys374 by NADPH oxidase–generated reactive oxygen species (ROS) disturbs actin polymerization in the lamellipodium of neutrophils, and either Cys374 mutation to Ser or S-nitrosylation impairs the formation of microfilaments in vitro [40,57,58]. Although structural constrains cannot be ruled out among the actions exerted by those modifications on actin polymerization and stability, prior studies have shown that mutation to Ser of Cys374 disturbs the binding of G-actin to profilin in vitro [40]. In cells, profilin–actin complexes prevent nucleation and spontaneous polymerization of actin at the pointed end of filaments, favoring their polymerization and treadmilling by providing monomeric actin to the barbed end of F-actin [59]. In this regard, our results show that profilin–actin complexes increased in T cells upon TCR triggering and that S-nitrosylation of β-actin on Cys374 disturbed its interaction with PFN1, suggesting a key role for this modification in the retrograde flow of actin that controls the localization and function of PKC-θ at the IS. We confirmed this possibility with the profilin-binding inactive mutant C374S, which hardly organized in F-actin bundles at the lamellipodium-like d-SMAC and activated but did not concentrate PKC-θ at the IS, whereas its corresponding PFN1-binding competent mutant C374A did, and was resistant to Cys-NO–induced PKC-θ dispersion.

A role for PFN1 in lamellipodium formation has been previously reported to be dependent on both poly-L-Pro- and actin-binding [60]. We have shown here that the actin-binding defective PFN1 mutant H119E disturbed the coalescence of PKC-θ at the c-SMAC and that WT PFN1 counteracted the dispersion of PKC-θ induced by Cys-NO. Our findings thus strongly suggested that, by organizing the lamellipodium-like d-SMAC, PFN1 regulated the localization of PKC-θ at the IS. Interestingly, a central role has been recently assigned to PFN1 as a master regulator of the major systems of actin polymerization in eukaryotic cells, favoring elongation of linear actin bundles by supplying profilin–actin complexes to both formins and Ena/vasodilator-stimulated phosphoprotein (VASP) but inhibiting nucleation and Arp2/3-mediated branching of actin by competing with WASP for the binding to ATP-activated G-actin [61,62]. In this regard, formin-mediated nucleation of linear actin bundles in the d-SMAC has been proposed to be of importance for the formation of lamellar actin arcs and the movement of signaling microclusters towards the c-SMAC; furthermore, a subset of actin filaments specifically organized via WASP regulates TCR-triggered distal signaling, making evident the presence of highly specialized functional actin structures in the IS [63,64]. It is thus feasible that the defective binding between S-nitrosylated β-actin and PFN1 may regulate the organization and activation of signaling microclusters at the IS by controlling the balance between actin structures resulting from the specific activities of formins and Arp2/3.

A dual role for localization and activation of PKC-θ at the IS in fostering effector T cell functions but disturbing the suppressor activities of regulatory T cells has been previously proposed [65]. Our loss-of-function studies and the actions exerted by the transnitrosylating agent Cys-NO on the localization and activation of PKC-θ at the IS of T cells are thus potentially relevant from a pathophysiological perspective. In this regard, recent interest has been given to mesenchymal stem cells (MSCs) and differentiated lymphoid stromal cells (e.g., fibroblastic reticular cells and lymphoid endothelial cells) as sources of iNOS-derived NO that can control T cell activation and differentiation in mice [66]. Nevertheless, rather than producing iNOS-derived NO, human MSCs seem to exert T cell suppression through prostaglandin E2 (PGE2) production and enzymatic depletion of tryptophan [67,68]. Although important differences between mice and humans should be taken into account before assigning cell sources and functions to NO in adaptive immune responses [69,70], previous studies have shown that NO from mouse lymphoid stromal cells disturbs the function of Th1 but not of Th2 cells [71]. Our study shows that eNOS-derived NO provides proactivation signals in human T cells, facilitating PKC-θ and NF-kB activation, a key signaling pathway for the differentiation and function of Th2 cells [16]. Whether exogenous NO from lymphoid stromal cells and endogenous NO from eNOS might coordinately regulate PKC-θ–dependent T cell fate decisions and functions at the IS warrants further investigation.

## Materials and methods

### Antibodies, cells, and reagents

Antibodies were sourced as follows: CD3ε OKT3 and CD19 mAbs (BioLegend); eNOS, CD7 and PKC-θ mAb (BD Biosciences); eNOS pAb and β-actin mAbs (Sigma); PKC-θ, and Lamin B1 pAbs (Santa Cruz Biotechnology); Profilin-1 mAb (Synaptic System); phospho-PKC-θ (Thr 538),–CARMA1 (Ser652), -IKKα (Ser176) /β (Ser177) pAbs, and IKBα and phospho–IKBα (Ser32/36) mAbs (Cell Signaling Technology); CARMA1 pAb (Abcam); golgin-97 mAb (Life Technologies) and goat anti-mouse (GAM) H&L chain-specific IgG (Merck Millipore). mCherry pAb was generously provided by Dr. J. M. Requena (CBMSO, Madrid, Spain)

The Raji lymphoblastoid B cell line was grown in RPMI 1640 medium (Gibco), supplemented with 8% FBS. CH7C17 Jurkat T cells transfected with a TCRvβ3 specific for a peptide from influenza hemagluttinin (HA) were grown in the presence of hygromycin (400 μg/ml) and puromycin (4 μg/ml) (Invivogen). Geneticin-418 (0.8 mg/ml, Invivogen) was also added for culturing CH7C17 T cells stably transfected with eNOS-, G2A-GFP, or mCherry-β-actin constructs. Human peripheral blood mononuclear cells were isolated from buffy coats from healthy donors (provided by “Centro de Transfusiones de la Comunidad de Madrid”) by separation on a biocoll gradient (Biochrom). After a 30-min plating step at 37°C, nonadherent cells were collected and cultured for 36 h in the presence of SEE (0.2 μg/ml) to induce lymphocyte proliferation. To obtain T lymphoblasts, IL-2 (50 U/ml) was added to the culture medium every 2 days. These cells were analyzed by flow cytometry, and their phenotype was 98% CD3, 48% CD4, 46% CD8, 0.5% CD56, 0.2% CD19, 0.5% CD14, 85% CD45RO, and 19% CD45RA. All experimental procedures involving primary human T cells were approved by the Ethics Committee of the CNB-CBMSO (CSIC).

S-nitroso-L-cysteine (Cys-NO) was prepared as described [72]. Other reagents were sourced as follows: Staphylococcal enterotoxin B and E (SEB and SEE) (Toxin Technology); poly-L-Lys and -Pro, Fibronectin, DNase I, 8Br-cGMP, ODQ, FBS, Polybrene, Sodium L-ascorbate, Cytochalasin D, Phalloidin-TRITC, and Nitro-L-Arg-methyl-ester (L-NAME) (Sigma); Phalloidin-Alexa488, -Alexa568, -Alexa647, and Cell Tracker Blue CMAC (Life Technologies); and Streptavidin-Sepharose (GE Healthcare Life Sciences).

### Plasmid and DNA constructs

Wild-type mCherry-β-actin was provided by Dr. M. Davidson (University of Florida, Tallahassee, Florida). Mutants of mCherry-β-actin on Cys257, Cys272, Cys285, and Cys374 were generated with the Quickchange site-directed mutagenesis kit (Agilent Technologies) and verified by DNA sequencing. The mutagenic primers GAGCGGTTCCGCTCCCCTGAGGCACTC (C257S), GCATGGAGTCCTCTGGC ATCCACG (C272S), CCATCATGAAGTCTGACGTGGACATC (C285S), GTCCAC CGCAAATCCTTCTAGGCG (C374S), GTCCACCGCAAAGCCTTCTAGGGATCC (C374A), GTCCACCGCAAAGACTTCTAGGGATCC (C374D) and their complementary oligonucleotides were used to generate the mCherry-β-actin Cys-to-Ser mutants 3C, 4C, C374S, C374A, and C374D. pLifeAct-EGFP was provided by Dr. M.A. Alonso (CBMSO, Madrid, Spain), Speckle mCherry-β-actin was from Dr. M. Vicente-Manzanares (Hospital Universitario de la Princesa, Madrid, Spain), pMSCV-PKC-θ-TagRFP-T was from Dr. M. Huse (Memorial Sloan-Kattering Cancer Center, New York, New York), pEGFP-PFN1 and -H119E were from Dr. P. Roy (University of Pittsburgh, Pittsburgh, Pennsylvania), and pcDNA3-CD28-CFP was from Dr. M. Bünemann (University of Würzburg, Würzburg, Germany).

### Cell transfection and flow cytometry

CH7C17 T cells (12 x 10^6^) were transfected with 25 μg of expression plasmids encoding wild-type (WT) mCherry-β-actin or its corresponding Cys mutants, using the Gene Pulser II (BioRad) at 350 V and 1000 μF. CH7C17 T cells stably transfected with the indicated protein constructs were selected by cell sorting with a FACSCVantage SE flow cytometer (BD Biosciences) and grown in the presence of 0.8 mg/ml G418.

For flow cytometry analysis of actin polymerization, eNOS and G2A cells were activated at 37°C on CD3 Ab-coated microwells (10 μg/ml) for the time indicated, detached gently with a pipette, fixed with 2% paraformaldehyde, permeabilized for 15 min with 0.3% saponine in PBS, and F-actin was stained with phalloidin-Alexa647. Mean fluorescence intensity of F-actin staining was analyzed with a FACSCanto flow cytometer (BD Biosciences) and FlowJo v7.6 software (TreeStar).

### CRISPR/Cas9-mediated genome editing

To generate eNOS KO cell lines, CH7C17 T cells were transfected with eNOS CRISPR/Cas9 KO plasmids encoding GFP, Cas9 nuclease, and eNOS single-guide RNAs for three 20 nt sequences, targeting exon 5 (TGGCTCAGGTGTTCGATGCC) and exon 6 (CTACGCGGGCTACCGGCAGC and AGCCCGCGTAGCGCACCAGC) (sc-400127, Santa Cruz Biotechnology), and sorted by flow cytometry using as a marker the fluorescence of GFP. To obtain individual clones, single cells were expanded. By western blot with eNOS-specific mAb, we identified two eNOS KO clones (KO1 and KO2) and also a control clone that expressed eNOS similarly as parental CH7C17 T cells. For validation of those clones at DNA level, exons 5 and 6 were amplified by PCR on genomic DNA extracts, using the external primers GTACTGGATACCAAGTCAGCTTC and CAGCCGTGCTGAATGCAGAG, followed by a nested PCR with the primers GAGACACCTGGCCCAG and GTCTCCGTGACCACC. PCR products were sequenced and, in some cases, cloned into pGEM-T easy before sequencing.

### Lentiviral shRNA interference

Viral particles containing eNOS-specific “MISSION shRNAs” (sheNOS1: TRCN0000045473 and sheNOS2: TRCN0000045477) in the lentiviral vector pLKO.1-Puro (Sigma) were produced at the CNIC Viral Vectors Unit. HEK293T cells were cotransfected (Lipofectamine 2000, Invitrogen) with control or eNOS shRNA pLKO.1-Puro, pCMV-dR8.91, and pVSV-G. Supernatants were collected after 48 h to 72 h and filtered (0.45 μm; Millipore). CH7C17 T cells were transduced with concentrated lentiviral vector stock at a multiplicity of infection of 10 in the presence of 8 μg/ml polybrene. Cells were centrifuged at 1,000 g for 90 min at 32°C and incubated at 37°C for 72 h.

### Immunofluorescence and confocal fluorescence microscopy

For the study of superantigen-specific T cell–APC conjugates, Raji APC (3 x 10^5^) were incubated for 20 min with 0.5 μg/ml SEB or SEE and then mixed with CH7C17 (4 x 10^5^) or human T lymphoblast (5 x 10^5^), respectively. Cells were then plated on poly-L-Lys–coated coverslips and incubated for 20 min at 37°C in humidified chambers. For studies of stimulation on activation surfaces, CH7C17 T cells (3 x 10^5^) were plated for 15 min onto coverslips treated with CD3 Abs. Cells were then fixed for 8 min in 2% paraformaldehyde in PBS at room temperature, rinsed with TBS pH 8.4, and then permeabilized for 30 s in 0.1% Triton-X100 before incubation with the corresponding Abs. eNOS was visualized by staining with a pAb, followed by anti-rabbit IgG Alexa647. The Golgi was stained with Golgin-97 mAb, followed by anti-mouse IgG Alexa488 (Life Technologies). PKC-θ was visualized by staining with a pAb, followed by anti-goat IgG Alexa488, Alexa555, or Alexa647 (Life Technologies). F-actin was stained with Phalloidin-Alexa488, -Alexa568, or -TRITC. Double immunofluorescence was performed using eNOS rabbit pAb and phalloidin-TRITC or PKC-θ goat pAb. Mounted coverslips were observed under a Zeiss LSM510 confocal laser scanning unit attached to a Zeiss Axio Imager.Z1 microscope equipped with a PL APO 63X/1.4 oil objective. Images stacks of 0.25, 0.34, or 0.40 μm were acquired with settings that in each case allowed the maximum signal detection below the saturation limits of the detectors. To assess the spatial distribution of PKC-θ in cells, the staining of PKC-θ accumulated as a major dot at the contact area of T cells with APCs was scrutinized through 15–18 confocal image sections and scored to obtain percentages of cells with PKC-θ at the c-SMAC, similarly as described previously [10]. The area and fluorescence of PKC-θ at the T-cell–APC contact site and the distance from eNOS were obtained with Leica image analysis software (Leica Microsystems) to process the image stacks acquired in the region of interest. For the study of the contact area between T and B cells, eNOS, G2A, or parental CH7C17 cells were allowed to conjugate with SEB-pulsed Raji B cells for 20 min. Cells were then fixed and labeled with anti-CD7 (Becton Dickinson) and Biotin anti-CD19 (Biolegend), subsequently stained with appropriate secondary anti-mouse (F(ab’)_2_) Alexa568 and Streptavidin Alexa 647, and mounted onto Prolong Gold-DAPI. Cells were studied by fluorescence confocal imaging. To analyze the distribution of CD7 and CD19 at the IS, three-dimensional reconstructions of the area were generated with the Leica software. The T cell–B cell contact surface was defined as the common area for CD7 and CD19. Area values were measured with a Matlab routine for analysis of SMACs regions from three independent experiments. Mean values from area were calculated. To analyze F-actin in phalloidin-TRITC–labeled T cell–APC conjugates, the mean fluorescence (AU) of cross-sections was drawn through a field, including the IS. To analyze F-actin and mCherry-β-actin levels in WT- and C374S-mCherry-β-actin T cells plated onto CD3 Ab-coated coverslips, we identify the region occupied by F-actin at representative lamellipodium-like d-SMACs of WT-mCherry-β-actin T cells using a median filter (with a kernel size of 20) and consecutively a thresholding on the resulting image. We identified in this way an internal and external area and calculated the average difference of the external minus internal radius. Then, in the three lower z-stack images, we identified just the external border and we shrank it in a way that the average difference of the radius was the mean value from WT-mCherry-β-actin T cells. We calculated F-actin and mCherry mean fluorescence intensity (MFI) per area inside and outside the region corresponding to the lamellipodium-like d-SMAC. This analysis was performed using a user-customized routine, written in Python, that uses image analysis and numerical calculus packages plus the packages for the graphical user interface.

For colocalization analysis of F-actin and eNOS, the nuclear region was excluded and a median filter applied to the raw images. To obtain the Manders colocalization coefficient, the JACoP plugin from ImageJ (NIH, http://rsbweb.nih.gov/ij/) was applied to filtered images.

In some cases, brightness and contrast adjustments with Photoshop (Adobe) were applied to images.

### Time-lapse confocal fluorescence microscopy

For time-lapse confocal fluorescence microscopy studies, T cells in phenol red-free RPMI-1640 medium containing 20 mM HEPES and 2% FBS or in Hank’s Balanced Salt Solution (HBSS) with 25 mM HEPES and 2% FBS were allowed to interact with SEB-pulsed Raji APC (1:1 cell ratio) on LabTec II chambers (Nalge Nunc International) or slides coated with FN (50 μg/ml) in Attofluor chambers (Invitrogen), maintained at 37°C in a 5% CO_2_ atmosphere, and observed under a confocal laser–scanning unit (TCS SP5; Leica) attached to an inverted epifluorescence microscope (DMI6000; Leica) fitted with an HCX PL APO 63X/1.40–0.6 oil objective. Fluorescence and differential interference contrast (DIC) frames were obtained simultaneously at 15 s, 30 s, or 45 s intervals. At least six confocal sections were necessary to capture the entire fluorescent signal. For F-actin and NO detection at the IS, CRISPR/Cas9 cells were transfected with LifeAct-EGFP and 24 h posttransfection were preloaded with the DAR-4M-AM probe (30 min, 37°C, 0.5 μM), extensively washed with complete medium, and resuspended in imaging medium. Stacks of 27–29 sections for fluorescence and bright field images were acquired at intervals of 17.54 s to 25 s. 3D analysis and maximal projections of the T cell–APC conjugates were generated to obtain a Z stack projection and the corresponding movies. Images were processed with accompanying confocal software (LCS; Leica), IMARIS, or WCIF ImageJ. Analysis of LifeAct-EGFP and NO densities of fluorescence at the IS was performed with the user-customized routine described above.

### TIRFM and image analysis

CH7C17, eNOS, and G2A T cells transfected with speckle mCherry-β-actin or pMSCV-PKC-θ-TagRFP-T were allowed to settle onto CD3 Ab-coated glass-bottomed microwell dishes (No 1.5, Mattek). Recording was initiated immediately after cell addition, and visualization was performed with an Andor-DU8285 VP-4094 camera coupled to a Leica AM TIRF MC M, mounted on a Leica DMI 6000B microscope. Images were acquired with a HCX PL APO 100.0x1.46 OIL objective and processed with the confocal software LCS (Leica). For β-actin speckle tracking and flow calculation and for PKCθ-mRFP recording, penetrance was 90 nm for 561 nm laser channel with the same objective angle (25 ms time exposure), and frames were taken every 260–500 ms. Synchronization was performed through the Leica software. Speed measures were obtained through the calculation of slopes from position versus time displacements, using a customized routine from Matlab software. To estimate the velocity of the speckles diffusing toward the center of the cell, we visualized the sum of the intensity time series. On these images the tracks of the diffusing speckles are emphasized, favoring the selection of a segment to identify the pixels explored by the speckle during its diffusion. A section of the whole stack is used to define a region of interest (ROI) around the wake of the speckle. We threshold this ROI and perform a linear fitting of the points. The velocity of the speckle is calculated from the slope given by the linear fit. The final slope represented for each cell corresponds to the mean from 16 different measurements out of the analysis of four sections depicted on the projections of the movie frames from the cells studied. ImageJ software (NIH) was used to analyze the corresponding movies.

### Cell fractionation in Triton X-100, immunoprecipitation, and western blotting

T Cells were lysed in buffer CSK (50 mM PIPES pH6.9, 50 mM NaCl, 5% glycerol, 5 mM EGTA, 5 mM MgCl_2_, 0.25 mM DTT, 0.1 mM ATP, 0.2% Triton-X100, 0.5 mM PMSF, 5 μg/ml aprotinin, and 1 μg/ml leupeptin). Cell extracts were incubated at 4°C for 20 min and centrifuged at 20,000 g for 15 min. Supernatants were collected as Triton X-100–soluble fractions. Proteins from Triton X-100–insoluble pellets were extracted in RIPA buffer (50 mM Tris-HCl pH7.4, 1% NP-40, 0.5% Na-deoxycholate, 0.1% SDS, 150 mM NaCl, 2 mM EDTA, 0.5 mM PMSF, 5 μg/ml aprotinin, and 1 μg/ml leupeptin), incubated for 15 min at 4°C, and centrifuged as before. For immunoprecipitation, protein extracts from parental and mCherry-transfected CH7C17 T cells (6 x 10^6^) were incubated overnight at 4°C with 3 μl anti-mCherry pAb and protein A-Sepharose beads. After three washes in lysis buffer, immunocomplexes were collected by centrifugation. Supernatants from either soluble or insoluble cell fractions, and immunocomplexes were resuspended in reducing Laemmli loading buffer and boiled for 5 min. Proteins were separated by SDS-PAGE, transferred to nitrocellulose, immunoblotted with specific Abs and imaged on a fluorescence scanner (Odyssey, LI-COR Biosciences) or chemiluminescence detection system (LAS-4000, GE Healthcare Life Science).

### Detection of NO production

Cells were stimulated at 37°C under continuous rotation with CD3 Ab or Raji B cells (cell ratio = 4:1) pulsed with SEB or SEE (1 μg/ml) in 1 ml HBSS. NO production was monitored for 30 min with an ISO-NOP NO electrode (World Precision Instruments), which was calibrated against known concentrations of NaNO_2_ under reducing conditions (KI/H_2_SO_4_) at 37°C.

### Construction, expression, and purification of His-tagged mCherry-β-actin and in vitro binding assay

6xHis-tagged WT, 3C, and 4C mCherry-β-actin were generated by subcloning WT-, 3C-, and 4C-mCherry-β-actin in the pET-His6TEV-LIC vector using the Ligase Independent Cloning (LIC) method. WT-, 3C-, and 4C-mCherry-β-actin were expressed in vitro in *Escherichia coli* translation lysates (New England Biolabs) supplemented with the eukaryotic chaperonin CCT/TRiC, following the protocol by Stemp et al. [42]. Briefly, CCT was purified from bovine testis similarly as described [73], and added to *E*. *coli* translation lysates (3 μM), to proceed with the in vitro transcription–translation reaction following the manufacturer’s protocol. To characterize the binding of soluble translated β-actins to DNase I, 0.2 μg of each one was diluted in 0.8 ml G-buffer (5 mM Tris-HCl pH 8.0, 0.2 mM ATP, 0.2 mM CaCl_2_) containing 0.1% Triton X-100 and incubated at room temperature for 1 h with DNase I covalently-linked to CNBr-activated Sepharose beads. After incubation, collected beads were washed three times in G-buffer containing 0.5% Triton X-100. DNase I-bound recombinant β-actins were eluted with reducing Laemmli buffer, separated by SDS-PAGE, and detected by immunoblot.

Translated actins were assayed for binding activity to profilin-1 as follows: recombinant mCherry-β-actin–coated agarose Ni^2+^ beads of 0.5 μg each were preincubated at room temperature in 150 μL G-buffer, containing or not containing 100 μM Cys-NO. After washing, 0.8 ml extracts from 6 x 10^5^ CH7C17 T cells in G-buffer containing 0.5% Triton X-100 and 0.5% BSA were added and incubated at 4°C for 30 min. Proteins bound to recombinant mCherry-β-actin–coated agarose Ni^2+^ beads were washed twice with G-buffer containing 0.1% Triton X-100, with the same buffer plus 5 mM imidazole, and proteins associated with recombinant β-actin eluted in Laemmli loading buffer. β-actin–bound profilin-1 was determinated by SDS-PAGE and immunobloting with a profilin-1–specific mAb.

### Biotin switch

Cells were treated with Cys-NO, stimulated with SEB-pulsed Raji cells or with CD3 Ab in the dark, and then lysed in buffer TENT (50 mM Tris pH 6.0, 1 mM EDTA, 0.1 mM neocuproine, 1% Triton-X100, 70 mM N-ethyl-maleimide (NEM), 0.5 mM PMSF, 5 μg/ml aprotinin, and 1 μg/ml leupeptin). After centrifugation of cell extracts, a blocking step was carried out in 2% SDS at 40°C for 30 min with agitation in the dark. Proteins were precipitated with acetone and resuspended in buffer HENS (100 mM HEPES pH 8.0, 1 mM EDTA, 0.1 mM neocuproine, and 2% SDS) containing 80 mM ascorbate and 0.4 mM biotin-HPDP; the samples were incubated for 60 min at room temperature with agitation. Proteins were reprecipitated with acetone and resuspended in 0.2 volume of HENS buffer and 0.8 volume of neutralization buffer (20 mM HEPES pH 7.7, 100 mM NaCl, 1 mM EDTA, and 0.5% Triton X-100). For precipitation of biotinylated proteins, samples were incubated with Streptavidin-Sepharose beads for 60 min at room temperature with agitation. Beads were washed three times with wash buffer (20 mM HEPES, 600 mM NaCl, 1 mM EDTA, and 0.5% Triton X-100) and once with elution buffer without β-mercaptoethanol (20 mM HEPES pH 7.7, 100 mM NaCl, and 1 mM EDTA). Proteins were eluted with elution buffer containing 200 mM β-mercaptoethanol for 20 min at 37°C with agitation. Supernatants were collected and proteins analyzed by immunoblot.

### Proteomic analysis

Proteomic analyses were performed similarly as previously reported [74]. Peptide mixtures were subjected to nano-liquid chromatography coupled to mass spectrometry for protein identification. Peptides were injected onto a C-18 reversed phase nano-column (75 μm I.D. and 50 cm, Acclaim-PepMap, Thermo) and analyzed in a continuous acetonitrile gradient, consisting of 0%–31% B in 130 min, 50%–90% B in 1 min (B = 98% acetonitrile, 0.1% formic acid). A flow rate of 200 nL/min was used to elute peptides from the RP nano-column to an emitter nanospray needle for real-time ionization and peptide fragmentation on an Orbitrap-ELITE mass spectrometer (Thermo). An enhanced FT-resolution spectrum (resolution = 120,000) followed by the CID-MS/MS spectra from the most intense fifteen parent ions were analyzed along the chromatographic run. Dynamic exclusion was set at 25 s.

For protein identification and characterization of IAM- and NEM-labeled peptides, fragmentation spectra were searched against MSDB database using the SEQUEST program. Two miscleavages were allowed, and an error of 15 ppm or 10 ppm was set for full MS or MS/MS spectra searches, respectively. All identifications were performed by Proteome Discoverer 1.0 software (Thermo). Decoy database search for FDR analysis was set at 0.05 by applying corresponding filters.

### Pull-down experiments with sepharose-coupled poly-L-Pro

Cells were centrifuged and resuspended in lysis buffer (25 mM Tris pH7.4, 150 mM NaCl, 1 mM EDTA, 5% glycerol, 1% Triton-X100, and protease inhibitors) at 4°C. Lysates were cleared by centrifugation and incubated (90 min, 4°C) with poly-L-Pro covalently linked to CNBr-Sepharose beads. After incubation, collected beads were washed three times with lysis buffer. PFN1 and β-actin were eluted with reducing Laemmli buffer and samples separated by 15% SDS-PAGE in parallel with corresponding whole cell lysates to detect PFN1 and β-actin inputs.

### Densitometry and statistical analyses

Quantitative estimation of proteins in immunoblots was performed by densitometric analysis of bands with ImageJ (NIH). Densitometric results were normalized to protein inputs and values processed with spreadsheet software (Microsoft Excel 2007) and represented as fold induction ratios (mean ± SEM). Data within groups were compared using analysis of variance (1-way ANOVA) with Newman-Keul’s posthoc correction for multiple comparisons or Student’s *t*-test to compare two sets of data (GraphPad, Prism 5). Statistical significance was set at a *p* value of 0.05 or lower.

## Supporting information

S1 Fig eNOS overexpression disturbs the coalescence of CD28 to the c-SMAC. Localization of CD28-CFP (cyan), PKC-θ (red), and eNOS- or G2A-GFP (green) at the IS of CD28-CFP-transfected eNOS and G2A T cells sorted by flow cytometry, and conjugated for 20 min with SEB-pulsed Raji APCs (asterisks). Bar = 4 μm. Percentages of cells with CD28 and PKC-θ concentrated at the c-SMAC are indicated as mean±SEM. n = 2. 94 G2A and 109 eNOS T cells were scored. † p≤0.05. On the bottom, X-Z plane projections of CD28 and PKC-θ at the IS, and fluorescence profiles along the yellow dotted lines are shown. Underlying data are provided in S1 Data.
(TIF)

S2 Fig eNOS knockdown and inhibition reduces NO production in T cells and concentrates PKC-θ at the c-SMAC. A) eNOS T cells were transduced with control or eNOS shRNAs; 72 h later, cells were conjugated for 20 min with SEB-pulsed Raji APCs (asterisks), fixed, stained for PKC-θ (red) and subsequently analyzed by confocal fluorescence microscopy. The fluorescence of eNOS-GFP (green) is also shown. Bar = 6 μm. On the right, percentages of cells with PKC-θ concentrated at the c-SMAC (upper) and the area it occupied at the IS (lower) are depicted. The mean±SEM of cell percentages and ±SD of areas from three independent experiments are shown. 113 control, and 141 eNOS shRNA cells were analyzed; *p≤0.05, ***p≤0.001. B) Electrochemical detection of NO production from SEE-specific primary human T lymphoblasts, eNOS, and CH7C17 T cells pre-treated or not with L-NAME (300 μM), and from eNOS T cells transduced with control or eNOS shRNAs, and mixed with non-pulsed-, SEB- or SEE-pulsed Raji APCs. NO synthesis at 30 min from 15x10^6^ cells is depicted. The mean±SEM is shown. n = 3. *p≤0.05, **p≤0.01, ***p≤0.001. C) SEE-specific primary human T lymphoblasts were pre-treated with the NOS inhibitor L-NAME (300 μM); 20 min later, cells were conjugated with SEE-pulsed APCs (asterisks), fixed, stained for PKC-θ (green) and analyzed by confocal fluorescence microscopy as in (A). Bar = 6 μm. On the right, the corresponding percentages of cells with PKC-θ concentrated at the c-SMAC, and the area occupied by PKC-θ are shown. The mean±SEM of cell percentages, and ±SD of areas are represented. n = 3. The area at the c-SMAC of 113 (untreated) and 175 (L-NAME-treated) cells was analyzed. *p≤0.05. Underlying data are provided in S1 Data.
(TIF)

S3 Fig eNOS does not change the area occupied by the T cell marker CD7 on the IS. A) CH7C17, eNOS, and G2A T cells conjugated with SEB-pulsed Raji B cells (ratio 1:1) for 20 min, showing the localization of CD7 (T cell) and CD19 (B cell). The T cell-APC contact area was determined by the co-distribution of both cell markers. On the right, 3D reconstruction of CD7 and CD19 at the IS of T cell-APC conjugates. Analyzed CH7C17, eNOS, and G2A T cells forming conjugates with Raji B cells were numbered. The fluorescence of eNOS and G2A (GFP, green) was superposed on bright field images (BF). Merge images for CD7 and CD19 are also included. Bar = 10 μm. B) The graph shows the distribution of calculated T cell-APC contact areas for each T cell type studied. The mean±SD is shown. n = 3. The area occupied by CD7 at the IS of 77 CH7C17, 75 eNOS, and 88 G2A T cells was studied. Underlying data are provided in S1 Data.
(TIF)

S4 Fig Characterization of eNOS KO CH7C17 T cells. A) On the top, the structure of human eNOS, showing the location of oxygenase and reductase domains, and the sites of myristoylation (M), and binding to Arg, haem, BH4, calmodulin (CaM), FMN, FAD, and NADPH. Modified from [75]. On the bottom, the 26 coding exons of eNOS gen with the targeted exons 5 and 6 (red) are depicted. B) A two strand representation of the DNA target sites in the exons 5 and 6 of eNOS (blue), the adjacent PAM (5’NGG) (green), and the 20 nt guide sequences at the 5’-end of the chimeric sgRNAs (red). C) Western blot analysis of eNOS and PKC-θ expression in parental, control, and eNOS KO1 and KO2 CH7C17 T cells. As control, protein extracts from HUVEC were loaded. n = 3. D) Electrochemical detection of NO production in parental, control, and eNOS KO1 and KO2 CH7C17 T cells stimulated with cross-linked CD3 Ab. NO synthesis at 30 min from 10 x10^6^ cells is depicted. The mean±SEM is shown. n = 4. **p≤0.01. E) Nested PCR of genomic DNA amplicons containing eNOS exons 5 and 6 from primary human T lymphocytes (Tlym), parental (CH7), control (C), and eNOS KO1 and KO2 CH7C17 T cells. F) Sequence alignment of exon 5 bases 4557–4605 from the NCBI reference sequence NC_018918.2 of eNOS human gene, a WT allele from control CH7C17 cells, and mutant alleles from eNOS KO1 identified by genomic PCR. The 20 nt DNA targets (blue), and the 3 nt PAMs (green) are shown. The adenine insertion in the exon 5 of eNOS KO1, leading to a premature TGA stop codon at T1187, is highlighted in red. Underlying data are provided in S1 Data.
(TIF)

S5 Fig PKC-θ phosphorylation in eNOS shRNA-transduced G2A T cells. PKC-θ Thr538 phosphorylation in G2A T cells transduced with control or eNOS shRNAs and stimulated 72 h later with SEB-pulsed Raji APCs for the time indicated. eNOS G2A-GFP is also shown. Normalized ratios of PKC-θ phosphorylation have been represented as mean±SEM. n = 4. Underlying data are provided in S1 Data.
(TIF)

S6 Fig Production of His-tagged recombinant WT-, 3C-, and 4C-mCherry-β-actin. A) Schematic representation of His-tagged WT-mCherry-β-actin and its corresponding Cys-to-Ser mutants 3C, and 4C generated in this study. The numbers identify the Cys on the C-terminal region of β-actin changed to Ser for each mCherry-β-actin mutant. B) SDS-PAGE Coomassie staining of proteins in fractions from the last purification step of the chaperonin CCT (Superose 6 column). The fractions labeled with asterisk were selected, mixed and used for the following experiments. C) In vitro synthesis of His-tagged recombinant WT-, 3C-, and 4C-mCherry-β-actin from *E*. *coli* translation lysates supplemented with CCT. Coomassie staining of the total protein fraction and immunoblot using mCherry-specific pAbs were performed in parallel. CCT-containing *E*. *coli* translation lysate without DNA template was loaded as control (“C”). The white asterisk labels protein bands corresponding to synthesized actins. BSA (1.5 μg) was also loaded. D) SDS-PAGE Coomassie staining of recombinant WT-, 3C-, and 4C-mCherry-β-actin in insoluble and soluble fractions from CCT-containing *E*. *coli* translation lysates. The white asterisk labels protein bands corresponding to synthesized actins. BSA (1.5 μg) was also loaded. E) Western blot analysis of recombinant WT-, 3C-, and 4C-mCherry-β-actin in soluble and insoluble fractions from *E*. *Coli* translation lysates in absence (N) or presence (CCT) of chaperonin. Recombinant actins were detected using mCherry-specific pAb. F) Binding of WT-, 3C-, and 4C-β-actin to DNase I. DNase I (DN) or control glycine (Gly) covalently linked to CNBr-activated Sepharose beads were incubated for 1 h at room temperature with recombinant WT-, 3C-, and 4C-mCherry-β-actin in pull-down assays. The SDS-PAGE gel was split in two pieces above the 37 kD molecular weight marker, and DNase I-bound β-actins were detected by immunoblot with mCherry-specific pAb whereas DNase I was Coomassie stained. Actin inputs are also shown.
(TIF)

S7 Fig Characterization of CH7C17 T cells stably transfected with WT-mCherry-β-actin or its corresponding Cys-to-Ser mutants. A) Flow cytometry analysis of WT-, 3C-, 4C- and C374S-mCherry-β-actin expression levels in stably transfected CH7C17 T cells. Underlying data are provided in S2, S3, S4, S5 and S6 Data, and files can be opened using FlowJo 10.2 software. B) CH7C17 T cells stably expressing WT-, 3C- and 4C-mCherry-β-actin were conjugated for 20 min with SEB-pulsed Raji APC (asterisks), fixed and then stained with PKC-θ pAb and anti-goat-IgG Alexa488 to analyze the localization of PKC-θ (green) and mCherry-β-actin (red) by confocal fluorescence microscopy. Bar = 4 μm.
(TIF)

S8 Fig Expression of C374S-mCherry-β-actin disturbs the coalescence of CD28 to the c-SMAC. Localization of CD28-CFP (cyan), PKC-θ (green) and WT- or C374S-mCherry-β-actin (red) at the IS of CD28-CFP-transfected WT-, and C374S-mCherry-β-actin CH7C17 T cells conjugated for 20 min with SEB-pulsed Raji APCs (asterisks). Bar = 4 μm. Percentages of cells with CD28 and PKC-θ concentrated at the c-SMAC are indicated as mean±SEM. n = 3. † p≤0.01. On the bottom, X-Z plane projections of CD28 and PKC-θ at the IS, and fluorescence profiles along the yellow dotted lines are shown. Underlying data are provided in S1 Data.
(TIF)

S9 Fig Expression and localization of C374A-, and C374D-mCherry-β-actin in CH7C17 T cells. A) Flow cytometry analysis of C374A-, and C374D-mCherry-β-actin expression levels in stably transfected CH7C17 T cells. Underlying data are provided in S7, S8 and S9 Data, and files can be opened using FlowJo 10.2 software. B) Localization of PKC-θ (green) and C374A- or C374D-mCherry-β-actin (red) in CH7C17 T cells conjugated with SEB-pulsed APCs (asterisks). Bar = 6 μm.
(TIF)

S1 Data Numerical values for graphs in figures. Figs 1A, 1B, 1C, 1E, 2A, 2B, 2C, 3A, 3B, 3C, 3D, 3E, 3F, 3G, 4B, 4C, 4F, 5A, 6A, 6C, 6D, 6E, 6F, 7A, 7B, 7D, 8C, 8D, 8F, 8G, 9E, 9F, 10C, 10E, 10F and 10G, S1, S2A, S2B, S2C, S3, S4D, S5 and S8 Figs.
(XLSX)

S2 Data Numerical Values for Flow Cytometry data in S7A Fig. Control CH7C17 parental cells.
(FCS)

S3 Data Numerical Values for Flow Cytometry Data in S7A Fig. WT mCherry-β-actin cells.
(FCS)

S4 Data Numerical Values for Flow Cytometry data in S7A Fig. 3C mCherry-β-Actin cells.
(FCS)

S5 Data Numerical Values for Flow Cytometry data in S7A Fig. 4C mCherry-β-Actin cells.
(FCS)

S6 Data Numerical Values for Flow Cytometry data in S7A Fig. C374S mCherry-β-Actin cells.
(FCS)

S7 Data Numerical Values for Flow Cytometry data in S9A Fig. Control CH7C17 parental cells.
(FCS)

S8 Data Numerical Values for Flow Cytometry data in S9A Fig. C374A mCherry-β-actin cells.
(FCS)

S9 Data Numerical Values for Flow Cytometry data in S9A Fig. C374D mCherry-β-actin cells.
(FCS)

S1 Movie F-actin and NO dynamics at the IS of control CH7C17 T cells. 3D reconstruction of the IS from confocal time-lapse Z-stacks from LifeAct-EGFP-transfected (green) CRISPR/Cas9 control T cells loaded with 0.5 μM the NO probe DAR-4M AM (red), and conjugated with SEB-pulsed Raji APCs. Time and Scale bar are indicated. Maximal projection method was used for reconstruction with Imaris software. Movie was mounted at 3 fps and corresponds to Fig 4A (Upper panels).
(AVI)

S2 Movie F-actin and NO dynamics at the IS of eNOS knock-out CH7C17 T cells. 3D reconstruction of the IS from confocal time-lapse Z-stacks from LifeAct-EGFP-transfected (green) CRISPR/Cas9 eNOS knock-out T cells loaded with 0.5 μM the NO probe DAR-4M AM (red), and conjugated with SEB-pulsed Raji APCs. Time and Scale bar are indicated. Maximal projection method was used for reconstruction with Imaris software. Movie was mounted at 3 fps and corresponds to Fig 4A (Bottom panels).
(AVI)

S3 Movie Comparative TIRFM of β-actin centripetal retrograde flow in eNOS and G2A T cells. Combined movies of time-lapse TIRFM of speckle mCherry-β-actin in eNOS and G2A T cells stimulated on CD3Ab-coated glass bottom chambers (No 1.5; Mat-Tek) at 37°C (5% CO2) in imaging medium (HBSS supplemented with 25 mM HEPES and 2% FCS). Images were taken at the rate of one frame per 410 ms with an Andor-DU8285 VP-4094 camera coupled to a Leica AM TIRF MC M mounted on a Leica DMI 6000B microscope (HCX PL APO 100.0x1.146 oil objective). Time is indicated in seconds. Bar = 10 μm.
(AVI)

S4 Movie TIRFM of PKC-θ microclusters in G2A T cells. Time-lapse TIRFM of G2A T cells transfected with PKC-θ-mRFP and stimulated on CD3Ab-coated glass bottom chambers (No 1.5; Mat-Tek) at 37°C (5% CO2) in imaging medium (HBSS supplemented with 25 mM HEPES and 2% FCS). Images were taken every 260 ms with an Andor-DU8285 VP-4094 camera coupled to a Leica AM TIRF MC M mounted on a Leica DMI 6000B microscope (HCX PL APO 100.0x1.46 oil objective). Time is indicated in seconds. Bar = 10 μm.
(AVI)

S5 Movie TIRFM of PKC-θ microclusters in eNOS T cells. Time-lapse TIRFM of eNOS T cells transfected with PKC-θ-mRFP and stimulated on CD3Ab-coated glass bottom chambers (No 1.5; Mat-Tek) at 37°C (5% CO2) in imaging medium (HBSS supplemented with 25 mM HEPES and 2% FCS). Images were taken every 500 ms with an Andor-DU8285 VP-4094 camera coupled to a Leica AM TIRF MC M mounted on a Leica DMI 6000B microscope (HCX PL APO 100.0x1.46 oil objective). Time is indicated in seconds. Bar = 10 μm.
(AVI)

S6 Movie Time-lapse confocal fluorescence microscopy of WT-mCherry-β-actin in the IS of CH7C17 T cells. CH7C17 T cells stably expressing WT-mCherry-β-actin (red) were conjugated with SEB-pulsed Raji APC at 37°C (5% CO2) in imaging medium (HBSS supplemented with 25 mM HEPES and 2% FCS) and images were taken every 30 s in an attofluor chamber with a HCX PL APO 63X/1.40–0.6 oil objective fitted on an inverted epifluorescence microscope attached to a confocal laser scanning unit (excitation 561nm; DMI6000 and TCS SP5; Leica). Bright field, fluorescence and merge images are shown. Time is indicated in seconds.
(AVI)

S7 Movie Time-lapse confocal fluorescence microscopy of WT-mCherry-β-actin in the IS of CH7C17 T cells treated with Cys-NO. CH7C17 T cells stably expressing WT-mCherry-β-actin (red) were conjugated with SEB-pulsed Raji APC and at 240 s, when the IS was established, 50 μM Cys-NO was added. Images were acquired at 37°C (5% CO2) in imaging medium (HBSS supplemented with 25 mM HEPES and 2% FCS) every 20 s in an attofluor chamber with a HCX PL APO 63X/1.40–0.6 oil objective fitted on an inverted epifluorescence microscope attached to a confocal laser scanning unit (excitation 561nm; DMI6000 and TCS SP5; Leica). Bright field, fluorescence and merge images are shown. Time is indicated in seconds.
(AVI)

S8 Movie Time-lapse confocal fluorescence microscopy of 3C-mCherry-β-actin in the IS of CH7C17 T cells. CH7C17 T cells stably expressing 3C mCherry-β-actin (red) were conjugated with SEB-pulsed Raji APC. Images were acquired at 37°C (5% CO2) in imaging medium (HBSS supplemented with 25 mM HEPES and 2% FCS) every 15 s in an attofluor chamber with a HCX PL APO 63X/1.40–0.6 oil objective fitted on an inverted epifluorescence microscope attached to a confocal laser scanning unit (excitation 561nm; DMI6000 and TCS SP5; Leica). Bright field, fluorescence and merge images are shown. Time is indicated in seconds.
(AVI)

S9 Movie Time-lapse confocal fluorescence microscopy of C374S-mCherry-β-actin in the IS of CH7C17 T cells. CH7C17 T cells stably expressing C374S-mCherry-β-actin (red) were conjugated with SEB-pulsed Raji APC. Images were acquired at 37°C (5% CO2) in imaging medium (HBSS supplemented with 25 mM HEPES and 2% FCS) every 15 s in an attofluor chamber with a HCX PL APO 63X/1.40–0.6 oil objective fitted on an inverted epifluorescence microscope attached to a confocal laser scanning unit (excitation 561nm; DMI6000 and TCS SP5; Leica). Bright field, fluorescence and merge images are shown. Time is indicated in seconds.
(AVI)

## Abbreviations

8Br-cGMP: 8-bromoguanosine 3',5'-cyclic monophosphate
APC: antigen presenting cell
Arp2/3: actin-related protein 2/3
CARMA1: CARD-containing MAGUK protein 1
Cas9: CRISPR associated protein 9
CCT: chaperonin containing TCP-1
cGMP: cyclic guanosine monophosphate
CRISPR: clustered regularly interspaced short palindromic repeats
c-SMAC: central supramolecular activation cluster
CTL: cytotoxic T lymphocyte
Cys-NO: S-nitroso-L-cysteine
DAG: diacylglycerol
d-SMAC: distal supramolecular activation cluster
EGFP: enhanced green fluorescent protein
eNOS: endothelial nitric oxide synthase
GFP: green fluorescent protein
HUVEC: Human umbilical vein endothelial cells
IAM: iodoacetamide
IκB: inhibitor of κB
IKK: IκB kinase
iNOS: inducible nitric oxide synthase
IS: immune synapse
KO: knockout
L-NAME: Nitro-L-Arg-methyl-ester
mRFP: monomeric red fluorescent protein
MSC: mesenchymal stem cell
MTOC: microtubule organizing center
NEM: N-ethyl-maleimide
NF-κB: nuclear factor kappa B
NO: nitric oxide
ODQ: 1H-[1,2,4]oxadiazolo[4,3-a]quinoxalin-1-one
PBL: peripheral blood lymphocyte
p-SMAC: peripheral supramolecular activation cluster
PGE2: prostaglandin E2
PFN1: profilin-1
PKC-θ: protein kinase C-θ
ROS: reactive oxygen species
SEB: staphylococcal enterotoxin B
SEE: staphylococcal enterotoxin E
sGC: soluble guanylate cyclase
shRNA: short hairpin RNA
SNARE: soluble NSF attachment protein receptor
TCR: T cell receptor
Th: T helper
TIRFM: total internal reflection fluorescence microscopy
TX-100: Triton X-100
VASP: vasodilator-stimulated phosphoprotein
WASP: Wiskott-Aldrich syndrome protein
WT: wild-type
