# Supplementary figures and images for: eNOS S-nitrosylates β-actin on Cys374 and regulates PKC-θ at the immune synapse by impairing actin binding to profilin-1
Source: PLoS Biol. 2017 Apr 10;15(4):e2000653. doi: 10.1371/journal.pbio.2000653 (PMC5386235; doi:10.1371/journal.pbio.2000653)

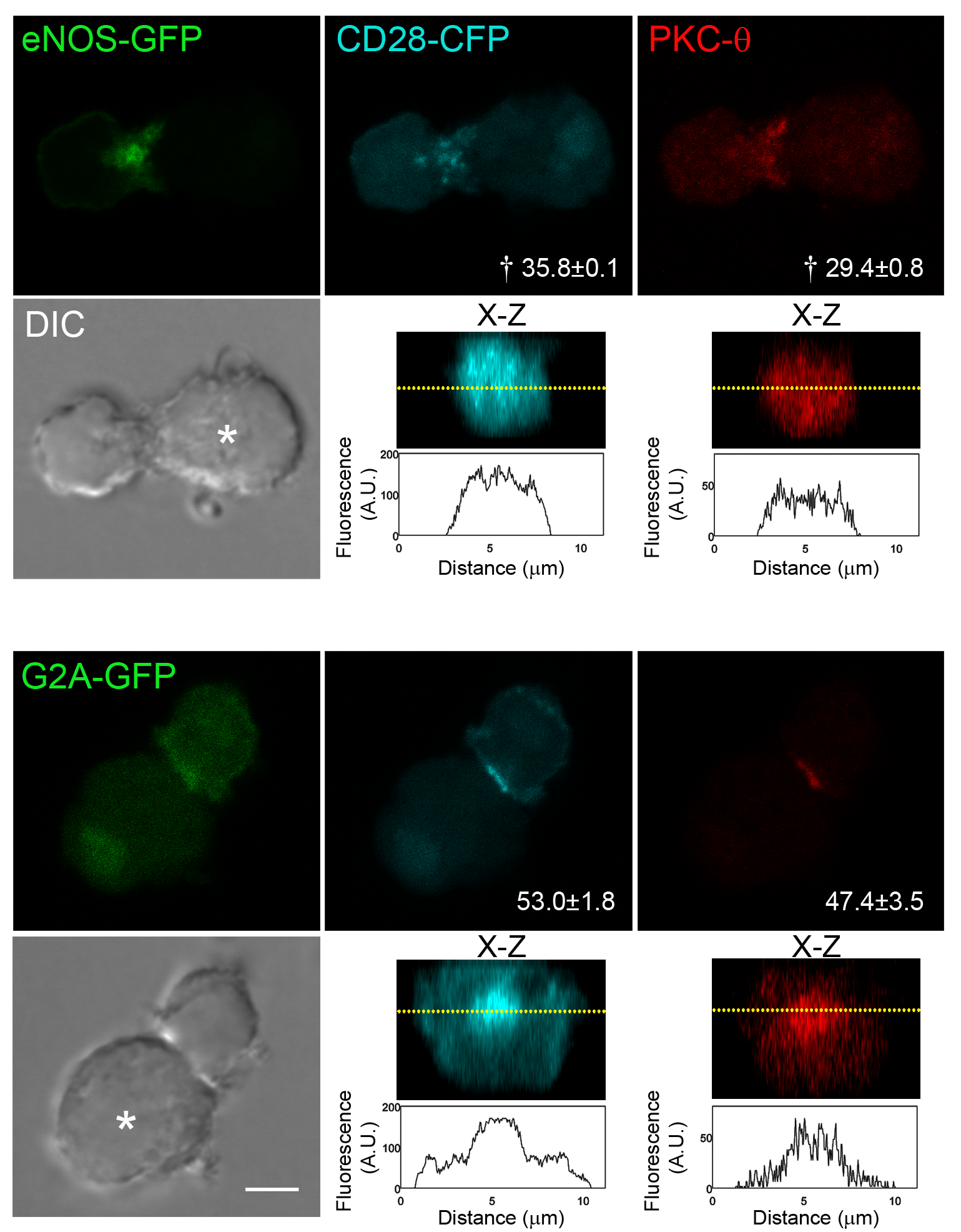

Supplement: S1 Fig — Localization of CD28-CFP (cyan), PKC-θ (red), and eNOS- or G2A-GFP (green) at the IS of CD28-CFP-transfected eNOS and G2A T cells sorted by flow cytometry, and conjugated for 20 min with SEB-pulsed Raji APCs (asterisks). Bar = 4 μm. Percentages of cells with CD28 and PKC-θ concentrated at the c-SMAC are indicated as mean±SEM. n = 2. 94 G2A and 109 eNOS T cells were scored. † p≤0.05. On the bottom, X-Z plane projections of CD28 and PKC-θ at the IS, and fluorescence profiles along the yellow dotted lines are shown. Underlying data are provided in S1 Data. (TIF) [file pbio.2000653.s001.tif]

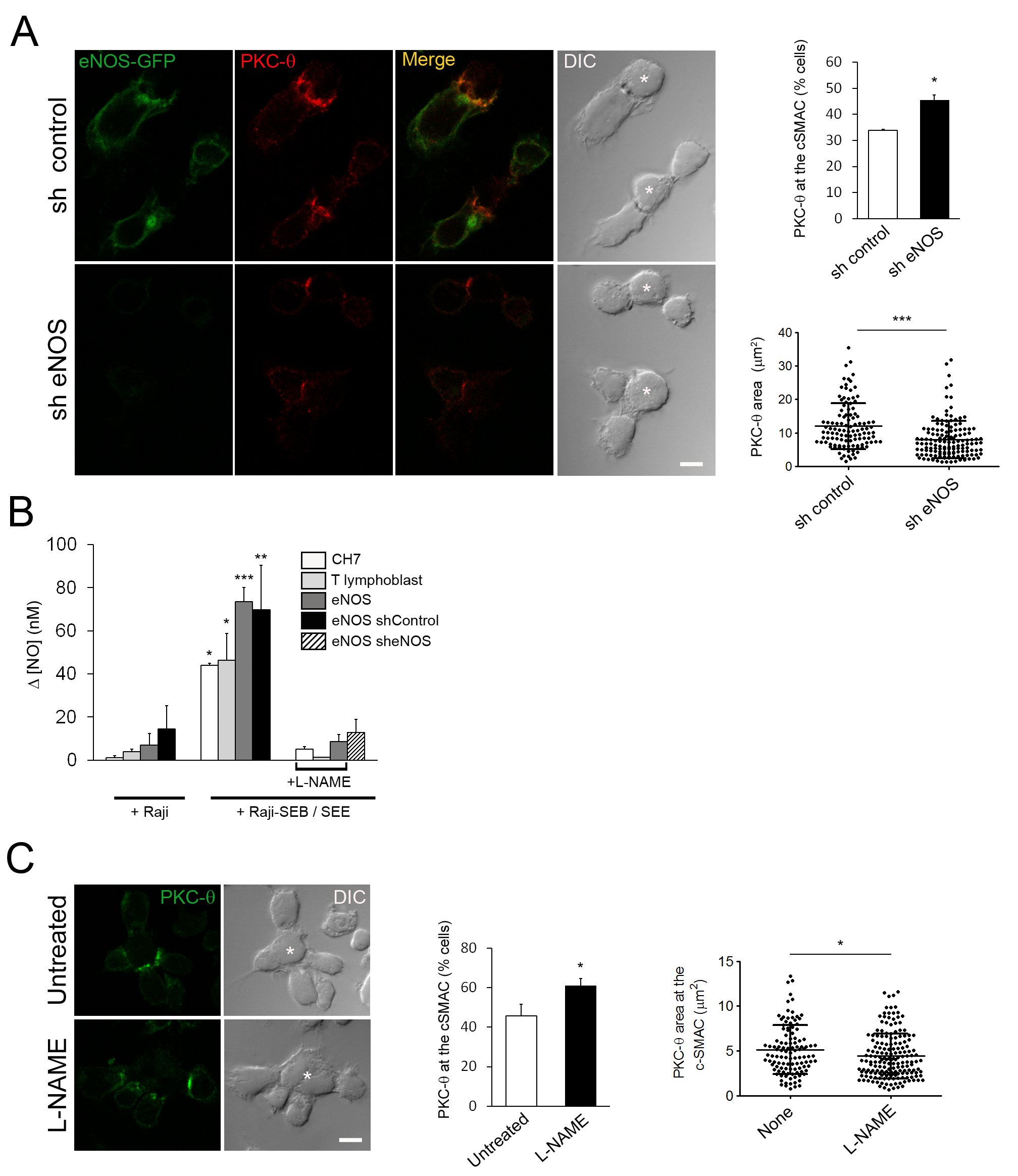

Supplement: S2 Fig — A) eNOS T cells were transduced with control or eNOS shRNAs; 72 h later, cells were conjugated for 20 min with SEB-pulsed Raji APCs (asterisks), fixed, stained for PKC-θ (red) and subsequently analyzed by confocal fluorescence microscopy. The fluorescence of eNOS-GFP (green) is also shown. Bar = 6 μm. On the right, percentages of cells with PKC-θ concentrated at the c-SMAC (upper) and the area it occupied at the IS (lower) are depicted. The mean±SEM of cell percentages and ±SD of areas from three independent experiments are shown. 113 control, and 141 eNOS shRNA cells were analyzed; *p≤0.05, ***p≤0.001. B) Electrochemical detection of NO production from SEE-specific primary human T lymphoblasts, eNOS, and CH7C17 T cells pre-treated or not with L-NAME (300 μM), and from eNOS T cells transduced with control or eNOS shRNAs, and mixed with non-pulsed-, SEB- or SEE-pulsed Raji APCs. NO synthesis at 30 min from 15x106 cells is depicted. The mean±SEM is shown. n = 3. *p≤0.05, **p≤0.01, ***p≤0.001. C) SEE-specific primary human T lymphoblasts were pre-treated with the NOS inhibitor L-NAME (300 μM); 20 min later, cells were conjugated with SEE-pulsed APCs (asterisks), fixed, stained for PKC-θ (green) and analyzed by confocal fluorescence microscopy as in (A). Bar = 6 μm. On the right, the corresponding percentages of cells with PKC-θ concentrated at the c-SMAC, and the area occupied by PKC-θ are shown. The mean±SEM of cell percentages, and ±SD of areas are represented. n = 3. The area at the c-SMAC of 113 (untreated) and 175 (L-NAME-treated) cells was analyzed. *p≤0.05. Underlying data are provided in S1 Data. (TIF) [file pbio.2000653.s002.tif]

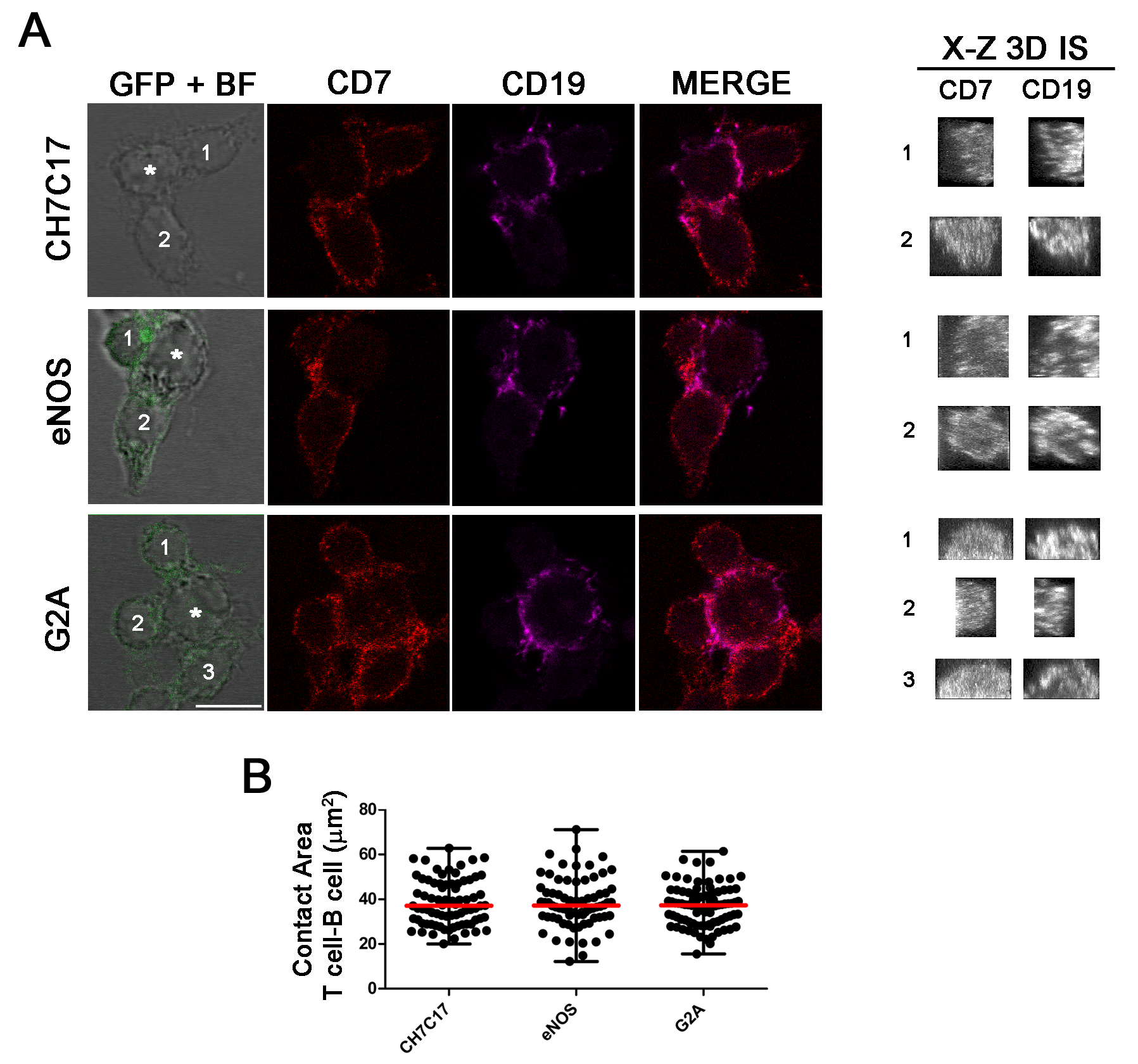

Supplement: S3 Fig — A) CH7C17, eNOS, and G2A T cells conjugated with SEB-pulsed Raji B cells (ratio 1:1) for 20 min, showing the localization of CD7 (T cell) and CD19 (B cell). The T cell-APC contact area was determined by the co-distribution of both cell markers. On the right, 3D reconstruction of CD7 and CD19 at the IS of T cell-APC conjugates. Analyzed CH7C17, eNOS, and G2A T cells forming conjugates with Raji B cells were numbered. The fluorescence of eNOS and G2A (GFP, green) was superposed on bright field images (BF). Merge images for CD7 and CD19 are also included. Bar = 10 μm. B) The graph shows the distribution of calculated T cell-APC contact areas for each T cell type studied. The mean±SD is shown. n = 3. The area occupied by CD7 at the IS of 77 CH7C17, 75 eNOS, and 88 G2A T cells was studied. Underlying data are provided in S1 Data. (TIF) [file pbio.2000653.s003.tif]

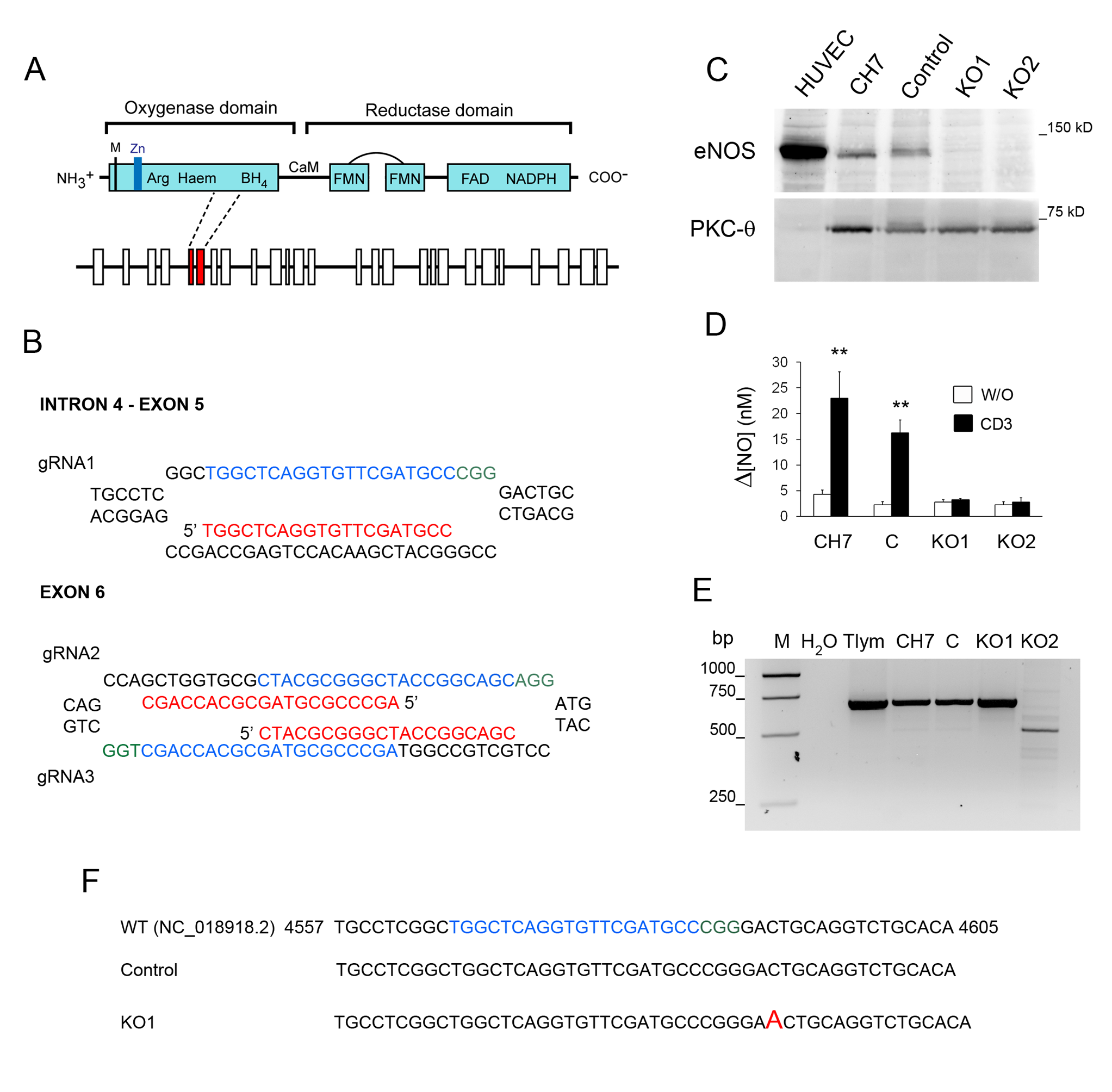

Supplement: S4 Fig — A) On the top, the structure of human eNOS, showing the location of oxygenase and reductase domains, and the sites of myristoylation (M), and binding to Arg, haem, BH4, calmodulin (CaM), FMN, FAD, and NADPH. Modified from [75]. On the bottom, the 26 coding exons of eNOS gen with the targeted exons 5 and 6 (red) are depicted. B) A two strand representation of the DNA target sites in the exons 5 and 6 of eNOS (blue), the adjacent PAM (5’NGG) (green), and the 20 nt guide sequences at the 5’-end of the chimeric sgRNAs (red). C) Western blot analysis of eNOS and PKC-θ expression in parental, control, and eNOS KO1 and KO2 CH7C17 T cells. As control, protein extracts from HUVEC were loaded. n = 3. D) Electrochemical detection of NO production in parental, control, and eNOS KO1 and KO2 CH7C17 T cells stimulated with cross-linked CD3 Ab. NO synthesis at 30 min from 10 x106 cells is depicted. The mean±SEM is shown. n = 4. **p≤0.01. E) Nested PCR of genomic DNA amplicons containing eNOS exons 5 and 6 from primary human T lymphocytes (Tlym), parental (CH7), control (C), and eNOS KO1 and KO2 CH7C17 T cells. F) Sequence alignment of exon 5 bases 4557–4605 from the NCBI reference sequence NC_018918.2 of eNOS human gene, a WT allele from control CH7C17 cells, and mutant alleles from eNOS KO1 identified by genomic PCR. The 20 nt DNA targets (blue), and the 3 nt PAMs (green) are shown. The adenine insertion in the exon 5 of eNOS KO1, leading to a premature TGA stop codon at T1187, is highlighted in red. Underlying data are provided in S1 Data. (TIF) [file pbio.2000653.s004.tif]

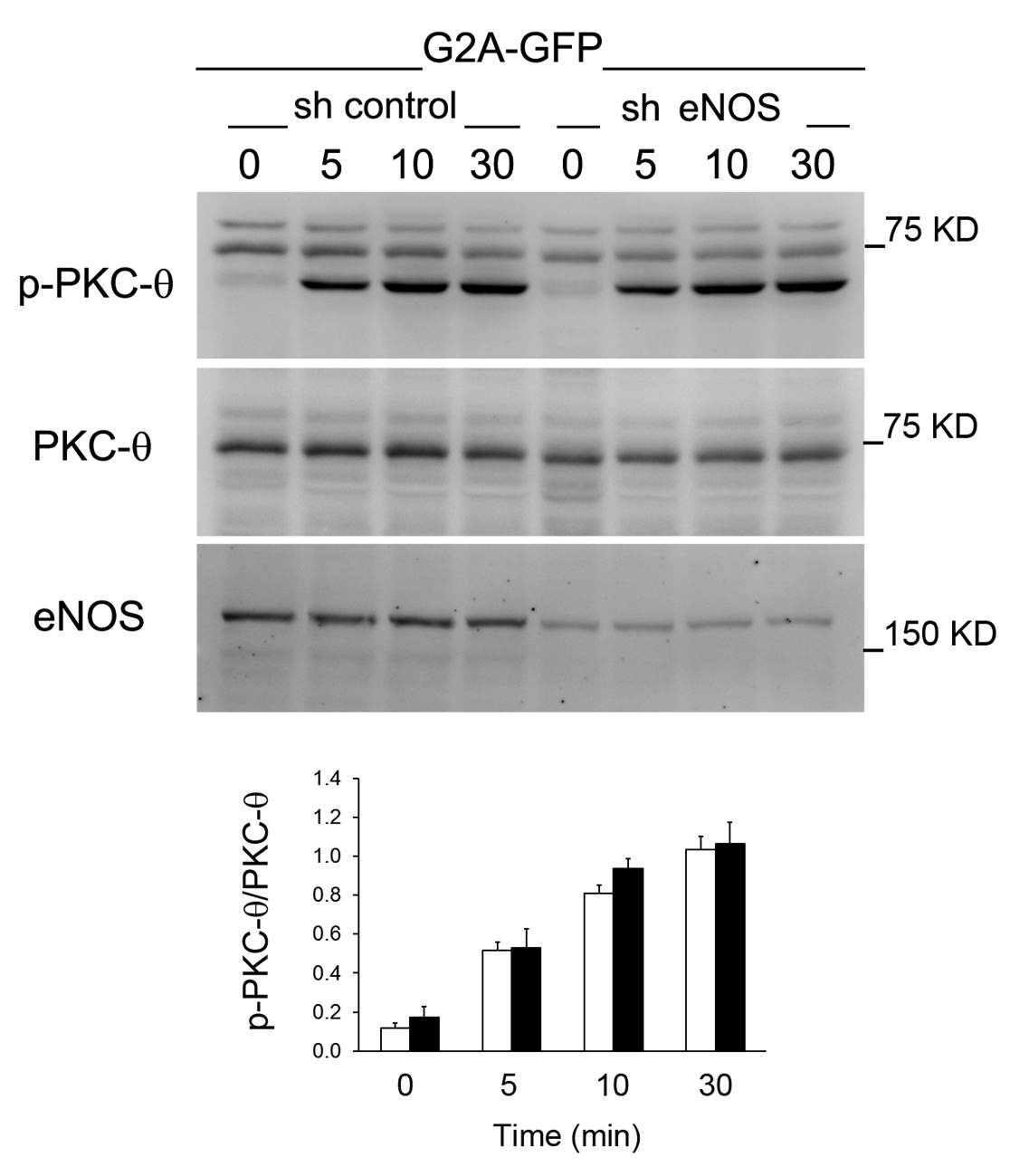

Supplement: S5 Fig — PKC-θ Thr538 phosphorylation in G2A T cells transduced with control or eNOS shRNAs and stimulated 72 h later with SEB-pulsed Raji APCs for the time indicated. eNOS G2A-GFP is also shown. Normalized ratios of PKC-θ phosphorylation have been represented as mean±SEM. n = 4. Underlying data are provided in S1 Data. (TIF) [file pbio.2000653.s005.tif]

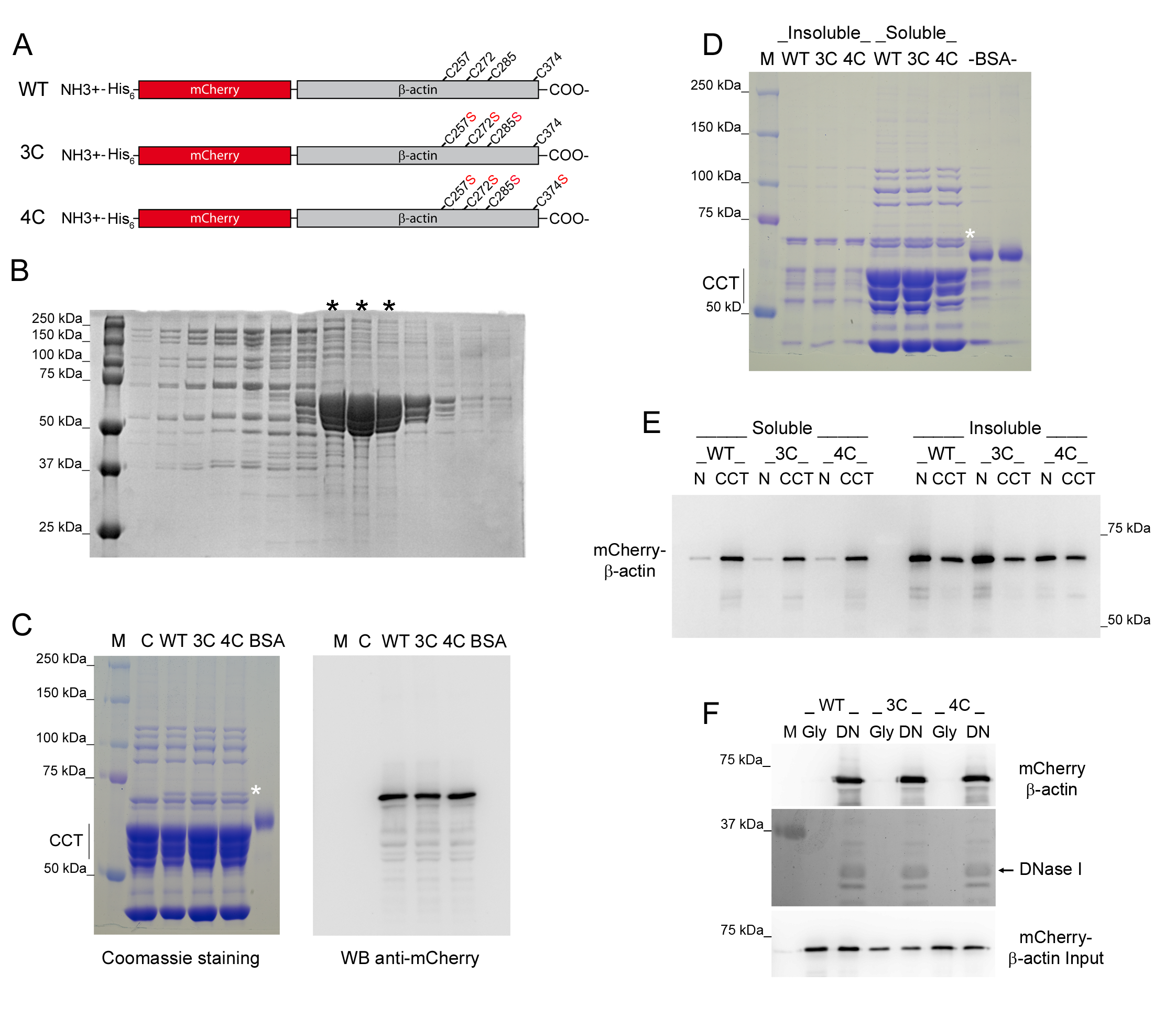

Supplement: S6 Fig — A) Schematic representation of His-tagged WT-mCherry-β-actin and its corresponding Cys-to-Ser mutants 3C, and 4C generated in this study. The numbers identify the Cys on the C-terminal region of β-actin changed to Ser for each mCherry-β-actin mutant. B) SDS-PAGE Coomassie staining of proteins in fractions from the last purification step of the chaperonin CCT (Superose 6 column). The fractions labeled with asterisk were selected, mixed and used for the following experiments. C) In vitro synthesis of His-tagged recombinant WT-, 3C-, and 4C-mCherry-β-actin from E. coli translation lysates supplemented with CCT. Coomassie staining of the total protein fraction and immunoblot using mCherry-specific pAbs were performed in parallel. CCT-containing E. coli translation lysate without DNA template was loaded as control (“C”). The white asterisk labels protein bands corresponding to synthesized actins. BSA (1.5 μg) was also loaded. D) SDS-PAGE Coomassie staining of recombinant WT-, 3C-, and 4C-mCherry-β-actin in insoluble and soluble fractions from CCT-containing E. coli translation lysates. The white asterisk labels protein bands corresponding to synthesized actins. BSA (1.5 μg) was also loaded. E) Western blot analysis of recombinant WT-, 3C-, and 4C-mCherry-β-actin in soluble and insoluble fractions from E. Coli translation lysates in absence (N) or presence (CCT) of chaperonin. Recombinant actins were detected using mCherry-specific pAb. F) Binding of WT-, 3C-, and 4C-β-actin to DNase I. DNase I (DN) or control glycine (Gly) covalently linked to CNBr-activated Sepharose beads were incubated for 1 h at room temperature with recombinant WT-, 3C-, and 4C-mCherry-β-actin in pull-down assays. The SDS-PAGE gel was split in two pieces above the 37 kD molecular weight marker, and DNase I-bound β-actins were detected by immunoblot with mCherry-specific pAb whereas DNase I was Coomassie stained. Actin inputs are also shown. (TIF) [file pbio.2000653.s006.tif]

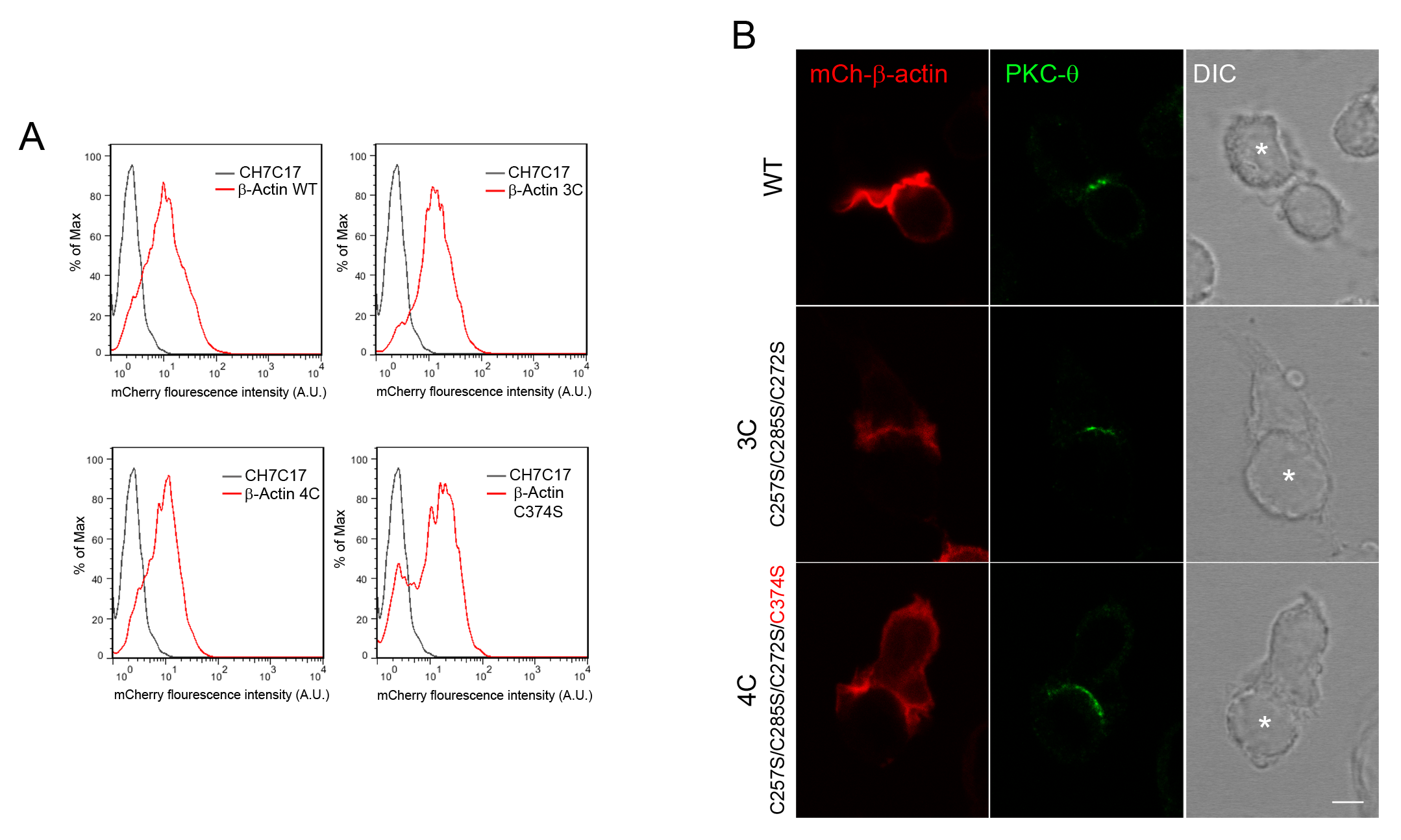

Supplement: S7 Fig — A) Flow cytometry analysis of WT-, 3C-, 4C- and C374S-mCherry-β-actin expression levels in stably transfected CH7C17 T cells. Underlying data are provided in S2, S3, S4, S5 and S6 Data, and files can be opened using FlowJo 10.2 software. B) CH7C17 T cells stably expressing WT-, 3C- and 4C-mCherry-β-actin were conjugated for 20 min with SEB-pulsed Raji APC (asterisks), fixed and then stained with PKC-θ pAb and anti-goat-IgG Alexa488 to analyze the localization of PKC-θ (green) and mCherry-β-actin (red) by confocal fluorescence microscopy. Bar = 4 μm. (TIF) [file pbio.2000653.s007.tif]

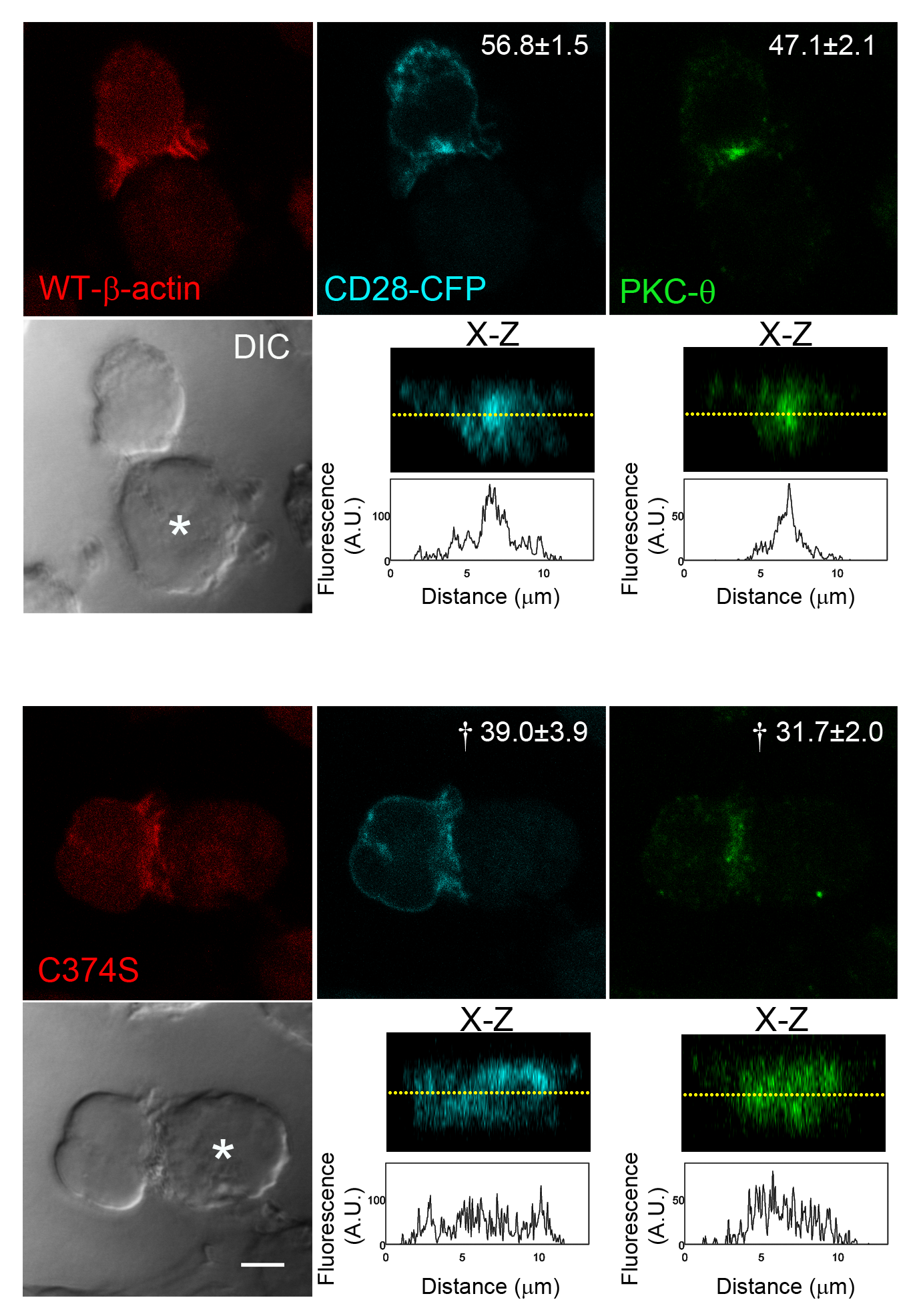

Supplement: S8 Fig — Localization of CD28-CFP (cyan), PKC-θ (green) and WT- or C374S-mCherry-β-actin (red) at the IS of CD28-CFP-transfected WT-, and C374S-mCherry-β-actin CH7C17 T cells conjugated for 20 min with SEB-pulsed Raji APCs (asterisks). Bar = 4 μm. Percentages of cells with CD28 and PKC-θ concentrated at the c-SMAC are indicated as mean±SEM. n = 3. † p≤0.01. On the bottom, X-Z plane projections of CD28 and PKC-θ at the IS, and fluorescence profiles along the yellow dotted lines are shown. Underlying data are provided in S1 Data. (TIF) [file pbio.2000653.s008.tif]

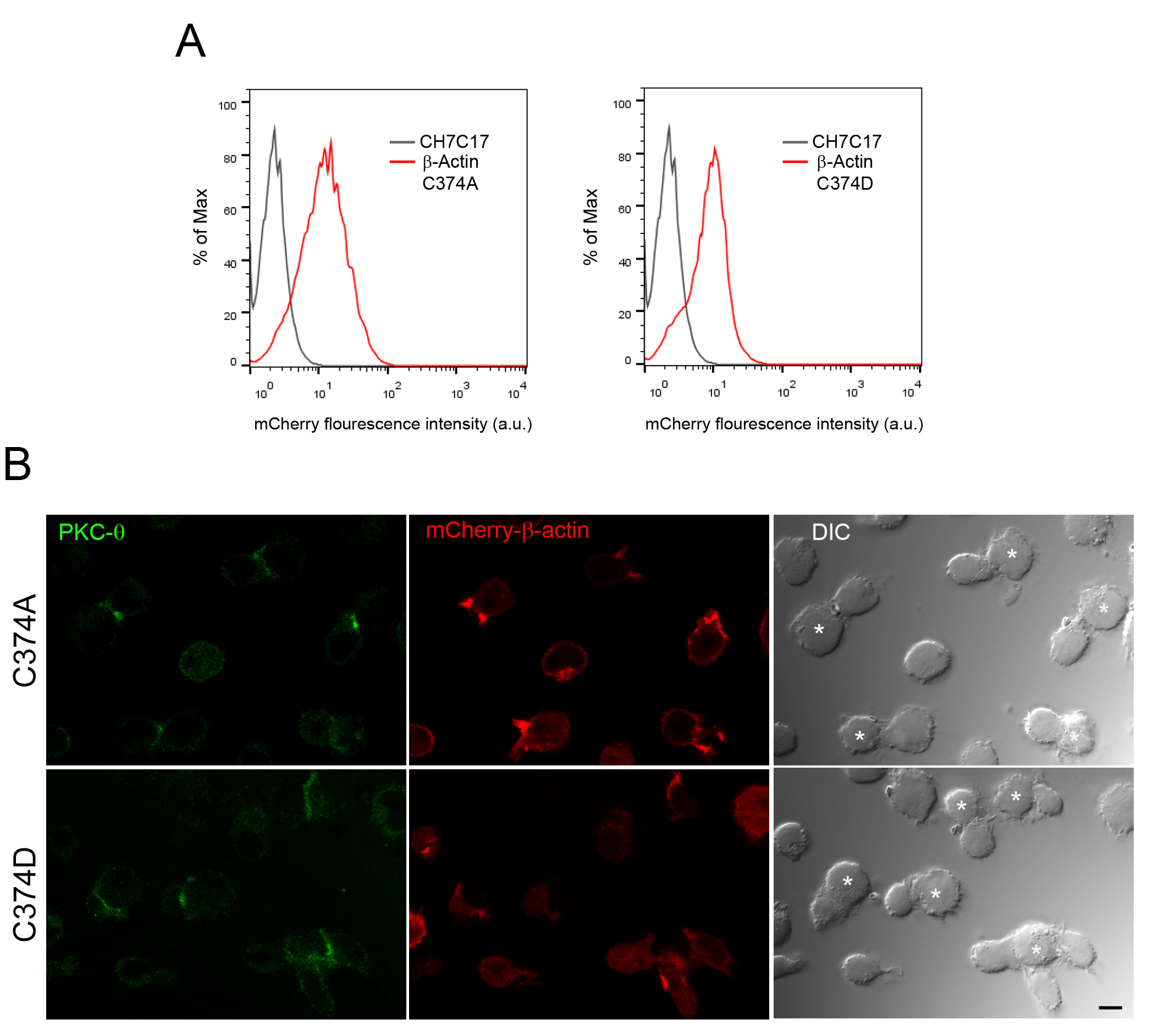

Supplement: S9 Fig — A) Flow cytometry analysis of C374A-, and C374D-mCherry-β-actin expression levels in stably transfected CH7C17 T cells. Underlying data are provided in S7, S8 and S9 Data, and files can be opened using FlowJo 10.2 software. B) Localization of PKC-θ (green) and C374A- or C374D-mCherry-β-actin (red) in CH7C17 T cells conjugated with SEB-pulsed APCs (asterisks). Bar = 6 μm. (TIF) [file pbio.2000653.s009.tif]
